# The Use of Google Trends in Health Care Research: A Systematic Review

**DOI:** 10.1371/journal.pone.0109583

**Published:** 2014-10-22

**Authors:** Sudhakar V. Nuti, Brian Wayda, Isuru Ranasinghe, Sisi Wang, Rachel P. Dreyer, Serene I. Chen, Karthik Murugiah

**Affiliations:** 1 Center for Outcomes Research and Evaluation, Yale-New Haven Hospital, New Haven, Connecticut, United States of America; 2 Yale School of Medicine, New Haven, Connecticut, United States of America; 3 Yale School of Public Health, New Haven, Connecticut, United States of America; University of Vienna, Austria

## Abstract

**Background:**

Google Trends is a novel, freely accessible tool that allows users to interact with Internet search data, which may provide deep insights into population behavior and health-related phenomena. However, there is limited knowledge about its potential uses and limitations. We therefore systematically reviewed health care literature using Google Trends to classify articles by topic and study aim; evaluate the methodology and validation of the tool; and address limitations for its use in research.

**Methods and Findings:**

PRISMA guidelines were followed. Two independent reviewers systematically identified studies utilizing Google Trends for health care research from MEDLINE and PubMed. Seventy studies met our inclusion criteria. Google Trends publications increased seven-fold from 2009 to 2013. Studies were classified into four topic domains: infectious disease (27% of articles), mental health and substance use (24%), other non-communicable diseases (16%), and general population behavior (33%). By use, 27% of articles utilized Google Trends for casual inference, 39% for description, and 34% for surveillance. Among surveillance studies, 92% were validated against a reference standard data source, and 80% of studies using correlation had a correlation statistic ≥0.70. Overall, 67% of articles provided a rationale for their search input. However, only 7% of articles were reproducible based on complete documentation of search strategy. We present a checklist to facilitate appropriate methodological documentation for future studies. A limitation of the study is the challenge of classifying heterogeneous studies utilizing a novel data source.

**Conclusion:**

Google Trends is being used to study health phenomena in a variety of topic domains in myriad ways. However, poor documentation of methods precludes the reproducibility of the findings. Such documentation would enable other researchers to determine the consistency of results provided by Google Trends for a well-specified query over time. Furthermore, greater transparency can improve its reliability as a research tool.

## Introduction

New tools are emerging to facilitate health care research in the Big Data era. One form of Big Data is that which accumulates in the course of Internet search activities. Internet search data may provide valuable insights into patterns of disease and population behavior.[1] In fact, the Institute of Medicine recognizes that the application of Internet data in health care research holds promise and may “complement and extend the data foundations that presently exist”.[2] An early and well-known example of utilizing Internet data in health has been the surveillance of influenza outbreaks with comparable accuracy to traditional methodologies.[3].

One tool that allows users to interact with Internet search data is Google Trends, a free, publically accessible online portal of Google Inc. Google Trends analyzes a portion of the three billion daily Google Search searches and provides data on geospatial and temporal patterns in search volumes for user-specified terms.[4] Google Trends has been used in many research publications, but the range of applications and methods employed have not been reviewed. Furthermore, there are no guidance or agreed standards for the appropriate use of this tool. A critical appraisal of the existing literature would increase awareness of its potential uses in health care research and facilitate a better understanding of its strengths and weaknesses as a research tool.

Accordingly, we performed a systematic review of the health care literature using Google Trends. To characterize how researchers are using Google Trends, we classified studies by topic domain and study aim. We conducted a subanalysis of surveillance studies to further detail their methods and approach to validation. We also assessed the reproducibility of methods and created a checklist for investigators to improve the quality of studies using Google Trends. Finally, we address general limitations in using Google Trends for health care research.

## Methods

### Overview of Google Trends

Google Trends provides access to Internet search patterns by analyzing a portion of all web queries on the Google Search website and other affiliated Google sites.[5] A description of the user interface is shown in Figure S1. Users are able to download the output of their searches to conduct further analyses.

The portal determines the proportion of searches for a user-specified term among all searches performed on Google Search. It then provides a relative search volume (RSV), which is the query share of a particular term for a given location and time period, normalized by the highest query share of that term over the time-series.[6], [7] The user can specify the geographic area to study, whether a city, country, or the world; data is available for all countries worldwide. Furthermore, the user can choose a time period to study, ranging from January 2004 to present, divided by months or days. The user is also able to compare the RSV of up to five different search terms or the RSV of a particular search term between geographic areas and between time periods. In addition, the user can choose from 25 specific topic categories to restrict the search, each with multiple sub categories for >300 choices in total, such as “Health → Mental Health → Depression”.

With respect to search input, multiple terms could be searched in combination with “+” signs and terms can be excluded with “-” signs. Quotations can be used to specify exact search phrases.[8].

### Study Selection

The review was conducted in accordance with PRISMA guidelines.[9] We included all studies that used Google Trends to answer research questions within the domain of health care. After an initial review, we included letters because they contained substantial original content. We also included studies using Google Insights for Search, a similar tool to Google Trends that was merged into Google Trends in 2012 (hereafter we will refer to studies using Google Insights for Search as using Google Trends for ease of reading).

We excluded studies that primarily focused on Google Flu Trends, a separate tool to specifically track seasonal variation in influenza trends. This tool is distinct from Google Trends and is therefore beyond the scope of this review. We also excluded articles that had no substantial use of Google Trends.

### Search Strategy

We identified relevant studies by searching Ovid MEDLINE (from inception to January 3, 2014) using a comprehensive search strategy. The list of subheadings (MeSH) and text words used in the search strategy for MEDLINE can be found in Appendix S1. We only included studies of humans written in the English language, and identified 1249 potential articles for inclusion. Since PubMed contains articles from life science journals in addition to articles indexed in MEDLINE, we conducted a search of PubMed (from inception to January 3, 2014) using a similar search strategy, but excluding the articles already identified from MEDLINE. This search identified an additional 871 potential articles, for a total of 2120 potential articles.

**Figure 1:**
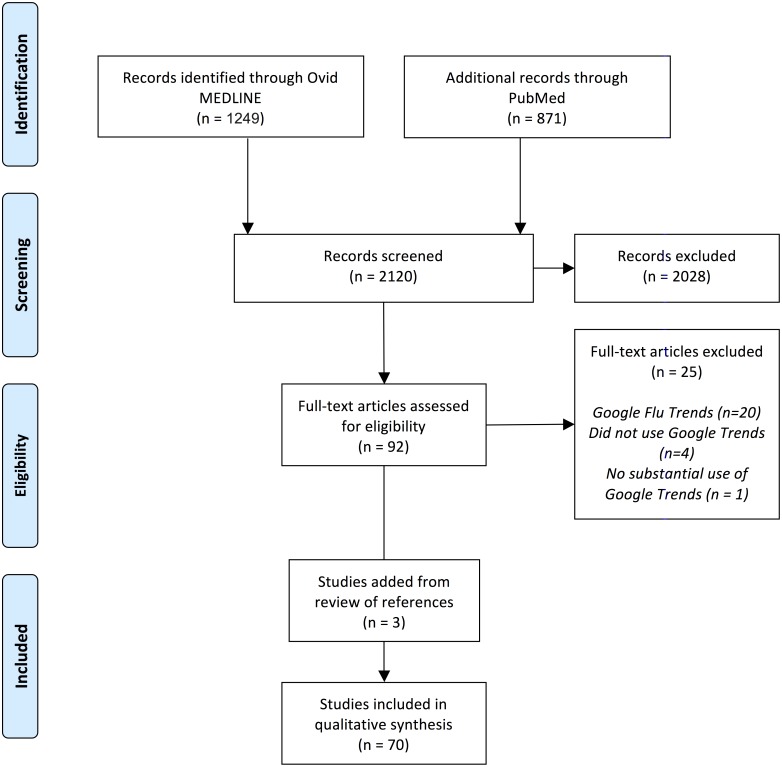
PRISMA Flow Diagram.

Two reviewers (S.V.N. and K.M.) independently reviewed the titles and abstracts of retrieved publications, and 92 articles met our inclusion criteria for full text review. We then excluded 25 studies that did not utilize Google Trends or that met at least one of our exclusion criteria (See Figure 1). We also included 3 articles found from the review of references.

The remaining 70 studies that met our inclusion criteria and did not meet our exclusion criteria form the studies included in this review. Data were abstracted from these studies using a standardized instrument, described below and in Table 1. All extractions were performed by at least two of the authors, and disagreements were resolved by consensus. We did not pool the results due to the heterogeneity of the articles, but we provide summary statistics.

**Table 1:** Variable Definitions.

VARIABLE	DEFINITION AND RATIONALE FOR SELECTION
**Purpose**	The primary aim of the study as derived from the introduction section of each paper. We abstracted the purpose of each study to provide the reader an overview of how researchers are using Google Trends and to facilitate the categorization of the study aims of Google Trends articles at large.
***Methods Variables*** *: We only searched within the methods section of each article for their methodology in using Google Trends. When the exact specification for each category was not in the methods, it was marked as not reported. When any of the specifications were not explicit or clearly evident, it was marked unclear. These variables were directly chosen based on the variables available within the Google Trends portal that could be manipulated by a user, namely location, query category, time period, and search input.*	
Location Searched	The area that was chosen to study.
Time Period Searched	The time period that was chosen to study; this was abstracted in as much detail (month, day) as provided in the article.
Query Category	The query category that was chosen. While the default category is “all categories” when using the tool, if this was not explicitly stated, this was marked as not reported.
Google Data Source	The use of Google Trends or Google Insights for Search. Google Insights for Search was merged into the Google Trends portal in 2012 and had similar capabilities as Google Trends.
Date of Access	The date that Google Trends or Google Insights for Search was accessed for use. This was looked for either in the methods or in the references of the paper. We abstracted this information because the Google Trends interface and its capabilities have changed over time, and it is important to have a full picture of what a researcher was able to search for at a given time.
*Search Input Variables: The search input is comprised of three components: terms utilized, the use of a combination of terms, and the use of quotation marks. Each component is important given how Google Trends works. Each search term input into Google Trends can be a single word or a combination of words (phrase). When a phrase is within quotation marks, the results will only include searches for the exact word order; otherwise, the results include searches of the words in any order. Multiple words in a search term can be separated by a ‘+’ sign, denoting an ‘OR’ function. On the other hand, a ‘-’ sign separating two words limits the results to searches containing the first word, but excludes searches with the second word. Thus, we chose these three components to abstract.*	
Terms utilized	The search terms that were input into the search bar to gain output. The exact terms utilized were abstracted, removing any quotation marks provided and other syntax, unless there were more than 15 terms, where only the number of terms was provided. This was a composite, where we abstracted all terms provided in the article and did not differentiate between different searches conducted.
Quotations used	The use of quotations if there are search terms within a search input that are greater than one word. If all the search inputs were single words, this was marked N/A. If there were no quotation marks provided, this was marked as not used. However, if quotation marks were used in the text, but not explicitly and clearly stated that they were used in the search input itself, this was marked unclear. For example, many articles used quotation marks to differentiate terms from the text of the paper but were unclear about use in the search input (e.g. We searched for “blue”, “blue dog”, and “red”).
Combination used	The use of “+” or “-“ marks for search inputs that utilize more than one term. When terms were stated to not be in combination, this was marked as not used. When this was not explicit, this was marked unclear. When there was only one term used for a search input, this field was marked N/A.
Clear Search Input	We defined clear search input as providing a clear use of quotes or combination when applicable.
Search Rationale	Reasoning provided for any part of the search input (terms used, syntax). If not provided, this was marked as not given. We abstracted this information because the rationale is necessary for a reader to better understand the study methods and to increase the face validity of the study.
**Analysis Type**	The type of analysis was designated as time trend (using Google Trends data for comparisons across time periods), cross sectional (using Google Trends data for comparisons across different locations at a single time period), or both. We noted all analyses using Google Trends data in the paper. We collected this variable to provide the reader a general perspective on how Google Trends data is being utilized for research.
**Primary Findings**	The main findings of the paper. If Google Trends-related results were not the primary finding, the findings associated with the tool was also included. We abstracted this information to easily provide the reader a means to understand what each study found.
**Number of Citations**	The number of citations for an article as determined by Google Scholar on March 9, 2013. We collected this information to assess the leveraging of Google Trends papers by the larger scientific community.
***Surveillance Variables:*** * For surveillance studies, we abstracted additional information, as the use of Google Trends data is more nuanced for these studies and the quality and reliability of a surveillance tool depends on methodological rigor and external validation.*	
Measure/outcome	The phenomena of interest in the study for which surveillance was intended. If multiple, all were noted. When not explicit, these were marked as not given.
Analysis Type	The analysis used for the surveillance portion of the study; not all analyses in the paper.
Time division	The quantum of time (e.g. weekly, monthly) for which Google Trends data was used in time trend analyses. For cross-sectional studies or those that did not use comparison data this was marked N/A.
Geographic division	The specific geographic area(s) studied.
Search terms used for surveillance	The specific search term(s) assessed for use in surveillance. Where multiple terms were used to produce a single set of predictions, the method by which these were combined is indicated. For example, some studies combined relative search volumes for different terms into a multivariate predictive model. This is distinguished from studies that used a single search volume – based on a combination of multiple search terms – for prediction.
Real-time or Lead-time	Whether Google Trends was used to track health phenomena in real time, to predict future patterns, or both. Real-time is using Google Trends data from a given time point/interval to serve as an alert for events occurring at the same time. Lead-time is using Google Trends data from a given time point/interval to predict events occurring at a later time.
Time horizon	The time period over which surveillance was assessed. This may differ from the dates input into Google Trends to extract data (as indicated in Table 2).
Comparison Data	For studies that used real-world comparison data for validation of Google Trends- based predictions, we extracted 1) the source of comparison data used; 2) the statistic used to measure the relationship between predictions and the gold standard; 3) the magnitude of relationship (e.g. correlation, r-squared) and corresponding p-values or confidence intervals (if reported); and 4) whether separate datasets (or time windows) were used for derivation of the predictive model (training) and assessment of its performance (testing).

### Evaluation of studies

#### Article Classification

To characterize how researchers are using Google Trends, we created a general descriptive classification of the articles according to their topic domain and study aim using an iterative process. The research team first worked together to examine all of the articles and identify common themes among the articles. We then assigned each article to the themes that emerged. After this initial step, we reassessed these groupings, refining the categorical domains and reassigning articles as needed, to create a classification construct that best characterizes the articles in the review. All disagreements during this process were resolved by consensus. This resulted in four topic domains (infectious disease, mental health and substance use, other non-communicable diseases, general population behavior) and three study aims (causal inference, description, surveillance). Of note, to categorize study aim, we examined the primary aim of the study as stated by the authors in the introduction of the paper. The definitions of these categories are described in the results.

#### Variable Abstraction

The variables extracted, along with the standard definitions and rationale for their selection, are listed in Table 1. These pertain to each study’s purpose, methodology (search variables, search input, and type of analysis), primary findings, and citations accrued.

We defined whether an article was “reproducible” based on whether the authors provided a clear documentation of all fields modifiable by the user, namely location of search, time period of search, query category, and terms utilized, as well as the clear documentation of combination used and quotations used when applicable. Only articles that clearly provided each of these fields (or were deemed not applicable for a given field(s) with all other fields provided) were defined as reproducible. We defined “clear search input” as providing a clear use of quotations and/or combination when applicable (see Table 1).

#### Subanalysis of Surveillance Studies

Following on the popularity of Google Flu Trends, there is particular interest in the potential use of Google Trends data to be operationalized as independent surveillance systems for other diseases. However, such surveillance systems require high standards of testing and validation before being deployed in the real world. Given these particular challenges, we performed a subanalysis of surveillance studies (as determined by study aim), abstracting additional information including the data sources used for validation, the strength of the relationship between predictions and external data, and other methodological characteristics listed in Table 1 and Table S1. We only assessed if validation data was used in surveillance studies, but we did not assess the quality of the validation process or data.

#### Assessment of Bias

Conventional tools to assess bias are largely limited to randomized trials and observational studies and are not readily applicable to studies using Google Trends data, which is observational in nature but does not involve individual research participants.[10], [11] Therefore, we attempted to address the two primary sources of potential bias within these studies: the search strategy and the validation of surveillance studies. Search methodology may introduce bias, as the selection of terms and changes in search input can alter results. We therefore captured all aspects pertinent to search strategy, including the provision of rationale for search input, for each article. The data sources and methods for validating findings in surveillance studies are also sources for bias, which we assessed in our subanalysis. We assessed for publication bias by evaluating the number of studies with positive findings versus neutral/negative findings.

## Results

### Study Sample

The 70 articles included in this systematic review are outlined in Table 2. Overall, 92% were original articles; the remaining were letters. Among the articles identified, we observed a seven-fold increase in publications utilizing Google Trends from 2009 to 2013 (Figure S2). Sixty-three percent of the articles chose to study areas outside of the United States alone. The median number (interquartile range) of article citations was 7 (1,16). The majority of studies (93%) presented positive findings with the tool compared to neutral/negative findings, indicating the possibility of publication bias.

**Table 2:** Study Characteristics.

						Search Method											
						Search Variables					Search Input			Rationale			
Topic Domain	Aim of Study	Author (Year)	Title		Purpose	Date of Access	Location Searched	Time Period Searched	Google Data Source	Query Category	Search terms used	Combination (Y/N)	Quotes (Y/N)	Rationale Provided for Search Input (Y/N)	Analysis	Primary Findings	Citations
Population Behavior	Causal Inference	Metcalfe (2011) [24]	Media coverage and public reaction to a celebrity cancer diagnosis		To investigated the impact of Jade Goody’s illness on media coverage of cervical cancer prevention, health information seeking behavior and cervical screening coverage.	Not reported	Not reported	June 1, 2008 to May 31, 2009	Google Insights	Not reported	Jade Goody, cervical cancer, smear test, human papilloma virus, HPV	Y	Unclear	N	Time Trend	A visual examination of the time plots suggests an association between the ‘Jade Goody’, ‘cervical cancer’ and ‘smear test’ search terms with clear peaks at, or just after, diagnosis, terminal phase and death. There were statistically significant cross correlation between JG and Cervical cancer searches and smear test searches but only weak correlation with HPV	23
Population Behavior	Causal Inference	Huang (2013) [25]	Assessing the impact of the national smoking ban in indoor public places in china: evidence from quit smoking related online searches		To investigate changes in online search behavior among Chinese Internet users in response to the adoption of the national indoor public place smoking ban	Not reported	China	January 1, 2009 to December 31, 2011	Google Trends	Not reported	Smoking ban, Quit smoking, Electronic cigarettes (In Chinese only)	N	Unclear	Y	Time Trend	The announcement and adoption of the indoor public place smoking ban in China generated significant increases in news coverage on smoking bans. There was a strong positive correlation between the media coverage of smoking bans and the volume of smoking ban(s), electronic cigarette, and Quit Smoking related search queries.	1
Population Behavior	Causal Inference	Ayers (2012) [26]	A Novel Evaluation of World No Tobacco Day in Latin America		To explore the potential of digital surveillance to evaluate the impacts of World No Tobacco Day on population awareness of and interest in cessation.	Not reported	Mexico, Colombia, Argentina, Peru, Venezuela, Chile, and Ecuador	2006 to 2011	Google Insights	Not reported	dejar de fumar (and variations)	N/A	Unclear	Y	Time Trend	Cessation news coverage and Queries indicative of cessation interest peaked around WNTD. A doubling in cessation news coverage was associated with approximately a 50% increase in cessation queries, suggesting that WNTD had a significant impact on popular awareness (media trends) and individual interest (query trends) in smoking cessation.	16
Population Behavior	Causal Inference	Ayers (2014) [27]	Do celebrity cancer diagnoses promote primary cancer prevention?		To explore the potential link between a public figure's cancer diagnosis and population primary cancer prevention, aiming to discover if such a link is plausible.	Not reported	Brazil	Not reported explicitly. A common period in October in each 2008 to 2011	Google Trends	Not reported	parar de fumar (and variations)	Unclear	Unclear	Y	Time Trend	Aggregating over the entire month (after Lula’s announcement), queries greatly increased for the 50 most common queries including the root terms “parar de fumar.”	1
Population Behavior	Causal Inference	Ayers (2011) [28]	Tracking the Rise in Popularity of Electronic Nicotine Delivery Systems (Electronic Cigarettes) Using Search Query Surveillance		To evaluate interest in electronic cigarette, particularly after interventions and in the presence of tough tobacco laws.	Not reported	United States, United Kingdom, Australia, Canada	January 2008 to September 2010	Google Insights	Not reported	66 terms	Y	Unclear	Y	Both	There is a tremendous increase in the popularity of ENDS, which has surpassed that of snus and NRTs. Stronger tobacco control, created by clean indoor air laws, cigarette taxes, and anti-smoking populations, were associated with consistently higher levels of ENDS searches.	79
Population Behavior	Causal Inference	Ayers (2011) [29]	Using Search Query Surveillance to Monitor Tax Avoidance and Smoking Cessation following the United States’ 2009 "SCHIP" Cigarette Tax Increase		To examine how smokers’ tax avoidance and smoking cessation internet search queries were motivated by the US 2009 state children’s health insurance program federal cigarette excise tax increase and two other state specific tax increases.	Not reported	United States (overall and by NY, Florida &Canada)	March 2007 to October 2010	Google Insights	Not reported	“Quit smoking” and “cheap cigarettes” were selected as the root terms for identifying search queries related to cessation and tax avoidance (used composite indicator)	Y	Unclear	Y	Time Trend	SCHIP tax was associated with an increase in cessation searches; however searches quickly abated and approximated differences from pre-tax levels in Canada during the months after tax. Tax avoidance increased during the months after tax compared to Canada, and trends were similar for US states.	18
Population Behavior	Causal Inference	Kostkova (2013) [30]	Major Infection Events Over 5 Years: How Is Media Coverage Influencing Online Information Needs of Health Care Professionals and the Public?		To investigate professional and public online information needs around major infection outbreaks and correlate these with media coverage.	January 4, 2012	Not reported	July 2006 to 2010	Google Trends	Not reported	Clostridium difficile, MRSA, tuberculosis, norovirus, influenza, meningitis.	N	Unclear	Y	Time Trend	Public information needs were more static, following the actual disease occurrence less than those of professionals, whose needs increase with public health events and the release of major national policies or important documents. Media coverage of events resulted in major public interest	1
Population Behavior	Causal Inference	Glynn (2011) [31]	The effect of breast cancer awareness month on internet search activity - a comparison with awareness campaigns for lung and prostate cancer		To assess the effects of the annual breast cancer awareness campaign on internet search activity, compare these effects with those of similar campaigns in prostate and lung cancer, and assess the overall levels of online activity relating to all 3 neoplasms between 2004–2009.	October 10, 2011	United States	January 2004 to December 2009	Google Insights	Not reported	Breast cancer, prostate cancer, lung cancer	N	Unclear	Y	Time Trend	There is a consistently higher level of background activity in breast cancer compared with that in lung and prostate cancer, and the October campaign stimulates online activity more effectively than equivalent campaigns for other malignancies.	11
Population Behavior	Causal Inference	McDonnell (2012) [32]	Should we fear "flu fear" itself? Effects of H1N1 influenza fear on ED use		To examine the effect of widespread public concern about flu on ED use.	Not reported	Not reported (one United States state)	2009. Compared three 1-week periods. But used the year data to identify the highest point.	Google Insights	Not reported	swine flu	N/A	Unclear	N	Time Trend	ED use and testing are high during fear week but with low admissions.	14
Population Behavior	Causal Inference	Ayers (2013) [33]	Digital Detection for Tobacco Control: Online Reactions to the United States’ 2009 Cigarette Excise Tax Increase		To analyze precise changes in Internet search queries around the SCHIP tax.	Not reported	United States	March 2007 to October 2010	Google Trends	Not reported	11 terms each for quit smoking and cheap cigarettes	Unclear	Unclear	Y	Time Trend	The SCHIP tax motivated specific changes in population considerations. Our strategy can support evaluations that temporally link tobacco control measures with instantaneous population reactions, as well as serve as a springboard for traditional studies, for example, including survey questionnaire design.	2
Population Behavior	Causal Inference	Reis (2010) [34]	Measuring the impact of health policies using Internet search patterns: the case of abortion		To study the impact of health policies across different regions in a more efficient and timely manner, with a focus on abortion.	Not reported	50 states in the United States and then 37 international countries	2004	Google Insights	Not reported	“abortion” in 19 languages.	N/A	N/A	N	Cross Sectional	Abortion rates globally are inversely proportional to abortion-related search volume, and directly proportional to laws.	13
Population Behavior	Causal Inference	Fenichel (2013) [35]	Skip the Trip: Air Travelers’ Behavioral Responses to Pandemic Influenza		To determine whether individuals engage in voluntary defensive behavior during an epidemic by estimating the number of passengers missing previously purchased flights as a function of concern for swine flu or A/H1N1 influenza using 1.7 million detailed flight records, Google Trends, and the World Health Organization’s FluNet data.	Not reported	Not reported	Not reported (April 1, 2008 to February 1, 2010 in figure)	Google Trends	Not reported	swine flu	N/A	Unclear	Y	Time Trend	Concern over ‘‘swine flu,’’ as measured by Google Trends, accounted for 0.34% of missed flights during the epidemic. The Google Trends data correlates strongly with media attention, but poorly (at times negatively) with reported cases in FluNet.	1
Population Behavior	Description	Stein (2013) [36]	Gauging interest of the general public in laser-assisted in situ keratomileusis eye surgery		To assess interest among members of the general public in laser-assisted in situ keratomileusis (LASIK) surgery and how levels of interest in this procedure have changed over time in the United States and other countries	Not reported	United States, United Kingdom, Canada, and India	January 1, 2007 to January 1, 2011	Google Trends	Not reported	LASIK, LASEK, Laser assisted in situ keratomileusis	N	Unclear	Y	Both	During 2007 to 2011, the Google query rate for “LASIK” was highest among persons residing in India, followed by the United Kingdom, Canada, and the United States, with the query rate declining everywhere but Canada. In all 4 of the US states examined, the query rate declined, and it declined further among US citizens after the Food and Drug Administration report release.	3
Population Behavior	Description	Ayers (2013) [37]	Seasonality in seeking mental health information on Google		To investigate seasonal patterns in mental health search queries to highlight their utility and help clarify the study of seasonality.	Not reported	United States and Australia	Not reported (appears 2006 to 2011 in figure)	Google Trends	Y (but used the category for determining initial terms, unclear whether subsequent searches were limited by categories)	adhd, anxiety, bipolar, depression, anorexia or bulimia, OCD, schizophrenia, suicide	Y	N/A	Y	Time Trend	Mental health queries across all illnesses/problems had pronounced peaks and troughs in the U.S. and Australian time series; similar in both countries; similar to Seasonal affective disorder	25
Population Behavior	Description	Murugiah (2010) [38]	Cardiopulmonary resuscitation (CPR) survival rates and Internet search for CPR: is there a relation?		To analyze the geographic variation in Google searches for the term “CPR” in cities with known OHCA survival rates to see if there is a relationship between them	Not reported	Regional areas within the United States	November 2005 to June 2010	Google Insights	Not reported	CPR	N/A	N/A	N	Cross Sectional	There was a significant correlation between relative search value and all rhythm OHCA. There was no correlation between area population and CPR relative search value.	1
Population Behavior	Description	Carr (2012) [39]	Search Query Data to Monitor Interest in Behavior Change: Application for Public Health		To assess patterns of public interest in major behavior change topics of ‘weight’, ‘diet’, ‘fitness’, and ‘smoking’ using publicly available search query data.	November 28, 2011	United States	January 4, 2004 to November 28, 2011	Google Insights	Not reported	weight, diet, fitness, smoking	Unclear	N/A	Y	Time Trend	There are significant, discernable temporal patterns of search activity for four areas of behavior change, with activity highest and January and declines thereafter.	4
Population Behavior	Description	Connolly (2009) [40]	What's on the mind of IVF consumers?		To understand changes in public interest in IVF over time and see whether there are any emerging trends.	Not reported	United States, United Kingdom	January 2004 to May 2009	Google Insights	Y – infertility, reproductive health	IVF, IVF Cost	N	Unclear	Y	Time Trend	Internet searches using IVF relative to searches within the infertility category remained unchanged in the USA, with a small decrease in the UK, and terms IVF and IVF cost have increased over the past 2 years. Inclusion of the term cost appears concentrated in the US states without insurance mandates.	7
Population Behavior	Description	Davis (2013) [41]	Using Google Trends to Assess Global Interest in 'Dysport (R)’ for the Treatment of Overactive Bladder		To develop a method for analyzing global Internet search activity for ‘Dysport’ specifically for the treatment of overactive bladder.	Not reported	Worldwide with sub regions	2004 to 2012	Google Trends	Not reported	dysport, overactive bladder	Unclear	Unclear	N	Both	Since 2009, mean global search activity for Dysport and overactive bladder has increased significantly on an annual basis	1
Population Behavior	Description	Bentley (2010) [42]	A rapid method for assessing social versus independent interest in health issues: A case study of ‘bird flu’ and ‘swine flu’		To analyze real-time online data, provided by the new Google Trends tool, concerning internet search frequency for health related issues, using bird flu and swine flu as a case study.	Not reported	Worldwide	April 24, 2009 to May 4, 2009 (obtained search data for swine flu and bird flu) & also collected data for bird flu from September 1, 2005 to December 30, 2005 (a period of genuine threat)	Google Trends	Not reported	Swine flu, bird flu	N	Unclear	N	Time Trend	The 2005 bird flu scare demonstrated almost pure imitation for 2 months initially, followed by a spike of independent decision, and for swine flu in 2009, imitation was the more prevalent throughout.	16
Population Behavior	Description	Liu (2012) [43]	Interest in Anesthesia as Reflected by Keyword Searches using Common Search Engines		To gauge general interest in anesthesia in comparison with surgery and pain using Internet keyword searches.	Not reported	Not reported	2004 to 2011	Google Insights	Not reported	anesthesia, anesthesia and safety, anesthesia side effects, anesthesiologist, surgery, surgeon, pain, pain after surgery, surgery pain.	N	N	N	Time Trend	Interest in surgery is constant, while in anesthesia it is decreasing. Side effects and surgical pain interest is increasing.	2
Population Behavior	Description	Hill (2011) [44]	Natural Supplements for H1N1 Influenza: Retrospective Observational Infodemiology Study of Information and Search Activity on the Internet		To identify and characterize websites that provide information about herbal and natural supplements with information about H1N1 and to examine temporal trends in the public’s behavior in searching for information about supplement use in preventing or treating H1N1.	Not reported	Not reported	January 1, 2009 to November 15, 2009	Google Trends	Not reported	Unclear	Unclear	Unclear	Y	Time Trend	A large number of websites support information about supplements and H1N1, and are less likely to be medically curated. Search activity for supplements was temporally related to H1N1/swine flu-related news reports and events.	6
Population Behavior	Description	Markey (2013) [45]	Seasonal variation in internet keyword searches: a proxy assessment of sex mating behaviors		To investigate seasonal variation in internet searches regarding sex and mating behaviors.	March 8, 2011	United States	January 2006 and March 2011	Google Trends	Not reported	47 terms	Unclear	Unclear	Y	Time Trend	Searches for pornography, prostitution, and mate-seeking can be explained by a 6-month cycle.	1
Population Behavior	Surveillance	Schuster (2010) [46]	Using Search Engine Query Data to Track Pharmaceutical Utilization: A Study of Statins		To examine temporal and geographic associations between Google queries for health information and healthcare utilization benchmarks.	November 26, 2009	United States	January 4, 2004 to June 28, 2009, June 2006 to June 2008	Google Trends	Not reported	Lipitor, simvastatin	N	N/A	Y	Both	Specific search engine queries for medical information correlate with pharmaceutical revenue and with overall healthcare utilization in a community.	8
Non-communicable Disease	Causal Inference	Davis (2012) [47]	Detecting internet activity for erectile dysfunction using search engine query data in the Republic of Ireland		To assess Internet search trends for erectile dysfunction (ED) subsequent to public awareness campaigns being launched within the Republic of Ireland, and whether the advent of such campaigns correlates with increased Internet search activity for ED.	Not reported	Ireland	January 2005 to December 2011	Google Insights	Y - All	erectile dysfunction	N/A	Unclear	N	Time Trend	Until 2007 no significant change in interest and then significant increase after campaign that year, with a similar trend in the number of web pages with information on ED	4
Non-communicable Disease	Description	Harsha (2014) [48]	Know Your Market: Use of Online Query Tools to Quantify Trends in Patient Information-seeking Behavior for Varicose Vein Treatment		To analyze Internet search data to characterize the temporal and geographic interest of Internet users in the United States in varicose vein treatment.	September 1, 2013	United States (regional data)	January 1, 2004, to September 1, 2012	Google Insights	Not reported	varicose vein treatment, varicose veins treatment, vein removal	Y	Unclear	Y	Both	Search traffic for varicose vein treatment increased significantly over the study period. May and June had higher searches, as did the southern United States.	0
Non-communicable Disease	Description	Breyer (2010) [49]	Use of Google in Study of Noninfectious Medical Conditions		To determine whether chronic noninfectious diseases with known variations in seasonal incidence (such as diabetes mellitus, blood pressure, myocardial infarction, and nephrolithiasis) would show seasonal variations in number of searches.	February 10, 2010	United States	February 2005 to January 2010	Google Insights	Not reported	diabetes, high blood pressure, hypertension,low blood pressure, hypotension, heart attack, myocardial infarction, kidney stones	Y	Unclear	N	Time trend	“Diabetes” had a sinusoidal pattern with a peak in March and a trough in August, “High blood pressure + hypertension” peaked during colder months, “low blood pressure + hypotension” peaked in the summer, “Heart attack + myocardial infarction” peaked in the winter months and declined in the summer, and “Kidney stones” peaked in the summer months.	11
Non-communicable Disease	Description	Leffler (2010) [50]	Frequency and seasonal variation of ophthalmology-related internet searches		To use internet search activity to reveal the intensity of public interest and seasonal variation in ophthalmology-related diseases, symptoms, and treatments.	Not reported	United States, United Kingdom, Canada, Australia	January 4, 2004 to October 19, 2008	Both	Not reported	38 terms related to eye, diabetes, controls: frostbite, heat stroke, sunburn, school	N	Unclear	Y	Time Trend	Internet ophthalmology searches relate (in decreasing order) to refractive correction, eye diseases, and eye symptoms. Search study reveals the seasonality and environmental associations of interest in health terms.	6
Non-communicable Disease	Description	Ingram (2013) [51]	Seasonal trends in restless legs symptomatology: evidence from Internet search query data		To utilize Internet search query data to test the hypothesis that restless legs symptoms vary by season, with worsening in the summer months	April 14, 2013	United States, Australia, Germany, Canada, United Kingdom	January 2004 to December 2012	Google Trends	Not reported	Restless legs	N/A	N	Y	Time Trend	Visual inspection of the search query data for the US and Australia revealed definite peaks and troughs. There were statistically significant seasonal effects found for restless legs in the US, Australia, Germany, and UK.	1
Non-communicable Disease	Description	Brigo (2014) [52]	Why do people Google epilepsy?: An infodemiological study of online behavior for epilepsy-related search terms		To evaluate changes in web search behavior occurring in English-speaking countries over time for terms related to epilepsy and epileptic seizures.	September 13, 2013	Not reported (Worldwide from abstract)	Not reported (January 2004 to September 2013 in abstract)	Google Trends	Y - health	epilepsy, seizure, seizures, SUDEP	N	N/A	N	Time Trend	Most people appear to use search engines to look for terms related to epilepsy to obtain information on seizure symptoms, possibly to aid initial self-diagnosis. Fears and worries about epileptic seizures and news on celebrities with epilepsy seem to be major factors that influence online search behavior.	2
Non-communicable Disease	Description	Bragazzi (2013) [53]	Infodemiology and infoveillance of multiple sclerosis in Italy		To assess Internet usage by MS patients, for seeking health and disease-related material for self-care and self-management purposes	Not reported	Italy	2004 to 2012	Google Trends	Not reported	sclerosi multipla	N/A	Unclear	N	Time trend	There were no cyclical trends yearly but long-term trends were present for MS searches. MS therapy and symptoms are the most searched MS-related terms.	1
Non-communicable Disease	Description	Braun (2013) [54]	Medical nowcasting using Google trends: application in otolaryngology		To evaluate the face validity of Google Trends by conducting searches related to otolaryngology within the German population.	Not reported	Germany	Not reported (2005 to 2012 from figure)	Google Trends	Not reported	sick listing, ENT doctor, tinnitus, cochlear implant, acute hearing loss, epistaxis, sinusitis	N	Unclear	N	Both	There is a seasonality in searching for sinusitis, which matches with searching for doctors.	0
Non-communicable Disease	Surveillance	Breyer (2010) [49]	Use of Google Insights for Search to Track Seasonal and Geographic Kidney Stone Incidence in the United States		To determine whether Internet search volume for kidney stones has seasonal and geographic distributions similar to known kidney stone incidence and can estimate disease burden.	May 10, 2010	United States (States and Metropolitan - NYC and Seattle)	January 2006 to December 2007 for national and regional. For Metropolitan – January 2005 to January 2010	Google Insights	Y - health but only for kidney stones	kidney stones, flank pain, kidney stone pain, kidney stone symptoms, kidney, passing kidney stone, California, textbook, hernia	Unclear	Unclear	Y	Both	The term “kidney stones” held the highest correlation with NIS monthly KS incidence, and GIS data were also correlated with regional NIS data.	21
Non-communicable Disease	Surveillance	Walcott (2011) [55]	Determination of geographic variance in stroke prevalence using Internet search engine analytics		To determine whether search engine query data can determine the prevalence of stroke.	Not reported	United States	January 1, 2005 to December 31, 2010	Google Insights	Y - All	stroke signs, stroke symptoms, mini stroke, heat	Y	N	Y	Cross Sectional	Query data allows for determination of relative stroke prevalence, albeit with a moderate correlation	6
Non-communicable Disease	Surveillance	Willard (2013) [56]	Internet Search Trends Analysis Tools Can Provide Real-time Data on Kidney Stone Disease in the United States		To evaluate the utility of using Internet search trends data to estimate kidney stone occurrence and understand the priorities of patients with kidney stones.	Not reported	United States	January 4, 2004 and April 10, 2010.	Google Insights	Not reported	Symptoms kidney stones, Kidney pain, Kidney stones pain, Kidney stone, Kidney stone causes, Kidney stone cause, Kidney infection, Kidney stones treatment. For analysis used “kidney stones”	N	Unclear	Y	Cross Sectional	Geographic and temporal variability in kidney stone disease appear to be accurately reflected in Internet search trends data, as the search volume index correlated significantly with established kidney stone epidemiologic predictors. The search term ranking suggested that Internet users are most interested in the diagnosis, followed by etiology, infections, and treatment.	10
Infectious Disease	Description	Johnson (2014) [57]	A comparison of Internet search trends and sexually transmitted infection rates using Google trends		To determine the relationship between sexually transmitted infection (STI)-related search engine trends and STI rates.	Not reported	States in the United States	2005 to 2011	Google Trends	Not reported	Gonorrhea symptoms, chlamydia symptoms, syphilis symptoms	N	Unclear	Y	Both	The term ‘‘chlamydia symptoms’’ was the most commonly searched of the 3 STI terms across all years. The frequency of the search terms relative to all other searches was greatest in states where STI rates are highest.	0
Infectious Disease	Description	Polkowska (2012) [58]	Increased incidence of Mycoplasma pneumoniae infection in Finland, 2010–2011		To assess the extent of ongoing epidemic in Finland, and whether changes in laboratory methods and practices and public interest in the epidemic during 2011 were related to the size of the epidemic.	December 21, 2011	Finland	2004 and 2011	Google Insights	Not reported	Mycoplasma	N/A	N/A	N	Time Trend	A high number of cases of *M. pneumoniae* infection especially during the current epidemic may partly reflect the intense public interest in and awareness of the disease.	23
Infectious Disease	Description	Seifter (2010) [59]	The utility of “Google Trends” for epidemiological research: Lyme disease as an example		To determine whether search volume for Lyme disease matches known seasonal and geographical characteristics of the disease.	July 16, 2009	United States	Not reported (appears to be 2004 to 2009 on examination of figure)	Google Trends	Not reported	Lyme disease, tick bite, cough	N	Unclear	N	Time Trend	Search traffic for the string “Lyme disease” reflected increased likelihood of exposure during spring and summer months; conversely, the string “cough” had higher relative traffic during winter months. The cities and states with the highest amount of search traffic for “Lyme disease” overlapped considerably with those where Lyme is known to be endemic.	38
Infectious Disease	Description	Rossignol (2013) [60]	A Method to Assess Seasonality of Urinary Tract Infections Based on Medication Sales and Google Trends		To determine if a seasonality of UTI exists or not and, if seasonality exists, to determine its magnitude.	June 5, 2013	Germany, France, Italy, United States, Australia, South Africa, China, and Brazil	January 11, 2004 to December 30, 2012	Google Trends	Not reported	cystitis, urinary tract infection (Queries were translated into the native language of the country)	Y	N	Y	Time Trend	An annual seasonality of UTIs was evidenced in seven different countries, with peaks during the summer.	1
Infectious Disease	Description	Mytton (2012) [61]	Influenza A (H1N1)pdm09 in England, 2009 to 2011: a greater burden of severe illness in the year after the pandemic than in the pandemic year		To compare the burden of influenza in the pandemic year 2009/10 with that in the year immediately after (2010/11) in England, and also assess public interest in influenza and antiviral usage over the same time period.	Not reported	England	2009 to 2010, 2010 to 2011	Not reported	Not reported	flu	N/A	N/A	N	Time Trend	There was a greater burden of severe illness in 2010/11 compared with 2009/10, but there was also much less public interest in influenza.	21
Infectious Disease	Surveillance	Jena (2013) [62]	Predicting New Diagnoses of HIV Infection Using Internet Search Engine Data		To determine if internet search queries could provide more up-to-date information about HIV	Not reported	State level in United States	2007–2010	Google Trends	Not reported	HIV	N/A	N/A	N	Cross Sectional	State Internet searches for “HIV” were highly correlated with state HIV incidence. Predicted rates of HIV incidence in 2009–2010 were also highly correlated with actual state estimates in those years	3
Infectious Disease	Surveillance	Zheluk (2013) [63]	Internet Search Patterns of Human Immunodeficiency Virus and the Digital Divide in the Russian Federation: Infoveillance Study		To assess whether online surveillance is a valid and reliable method for monitoring HIV in the Russian Federation.	Not reported	Russia	2011	Google Trends	Not reported	HIV, AIDS (in Russian)	N	N/A	Y	Cross Sectional	On a nationwide analysis, “HIV” and “AIDS” search volumes both correlated with HIV prevalence across regions. However, Google data are not adequate for *subnational* HIV surveillance in Russia, as data are unavailable for many regions.	1
Infectious Disease	Surveillance	Althouse (2011) [64]	Prediction of Dengue Incidence Using Search Query Surveillance		To predict dengue incidence using search query data	February 18, 2011 for Singapore and March 2, 2011 for Bangkok	Singapore & Bangkok	2004 to 2011	Google Insights	Not reported	Dengue (3 different languages). Final AIC model included 13 terms for Singapore and 7 terms for Bangkok.	Unclear	Unclear	Y	Time trend	Specific internet search terms are highly correlated with Dengue incidence	29
Infectious Disease	Surveillance	Carneiro (2009) [65]	Google Trends: A Web-Based Tool for Real-Time Surveillance of Disease Outbreaks		To introduce the more generic Google Trends (GT) tool to health professionals, to show how they can track disease activity of interest to them	October 1, 2009	United States	January 2004 to October 2008 in figure	Google Trends	N (but reported in figure)	West Nile virus, fever, headache, fatigue, rash, eye pain, rsv, bird flu	N	Unclear. Used brackets	N	Both	In the examples of WNV and RSV given above, the data show good correlation to seasonal spikes in disease activity. The example of “bird flu” showed spikes in search volume in regions where there were no actual cases of disease	123
Infectious Disease	Surveillance	Samaras (2012) [66]	Syndromic surveillance models using Web data: The case of scarlet fever in the UK		To apply data from Web search queries from Google Insights for Search for the surveillance of scarlet fever in the UK using two statistical methods.	Not reported	United Kingdom	2008 to 2010	Google Insights	Y	25 sets of searches for scarlet fever	Y	Unclear	Y	Time Trend	The peak and the spread of scarlet fever were predicted 5 weeks before the arrival of the peak.	1
Infectious Disease	Surveillance	Kang (2013) [67]	Using Google Trends for Influenza Surveillance in South China		To examine the temporal correlation between Google Trends related to influenza and conventional surveillance data in Guangdong province to determine if an increase of web search matches actual influenza activity in this province.	Not reported	Guangdong province, China	2008 to 2011	Google Trends	Not reported	Flu, Common cold, Fever, Cough, Sore throat, Influenza A, H1N1. (Every selected term consisted of one translated word and its synonyms in the Chinese language.)	N	N	Y	Time Trend	Correlations between ILI and influenza virus surveillance and Google Trends varied based on the Google search terms used, with the strongest correlation for fever. When compared with influenza virological surveillance, the term Influenza A had a statistically significant correlation coefficient.	7
Infectious Disease	Surveillance	Pelat (2009) [68]	More Diseases Tracked by Using Google Trends		To develop a surveillance approach to non-influenza diseases by examining the relationship between search engine query data with diseases other than influenza and in languages other than English.	February 27, 2009	France	January 2004 to February 2009	Google Insights	Not reported	grippe, aviaire, vaccin, gastro-enterite, gastroenterite, gastroenterite, gastroenterite, gastro enterite, gastro enterite, varicelle	Y	Unclear. Used brackets	Y	Time Trend	Robust Influenza search correlated with data; gastroenteritis correlated well with data, as did chickenpox searches; lag time of 0 weeks best for influenza-like illnesses, while one week lag time best for chickenpox. For each of 3 infectious diseases, 1 well-chosen query was sufficient to provide time series of searches highly correlated with incidence.	69
Infectious Disease	Surveillance	Desai (2012) [69]	Norovirus Disease Surveillance Using Google Internet Query Share Data		To assess whether Internet search trends are appropriate for monitoring norovirus disease	Not reported	United States	January 1, 2004 and April 30, 2010	Google Insights	Y - Gastro Esophageal Reflux Disease and Digestive Disorders	norovirus, vomiting,diarrhea, nausea, abdominal pain, stomach virus, food poisoning, gastroenteritis, Norwalk virus, rotavirus. Considered additional search terms strongly correlated with these	Y	Unclear. Used brackets	Y	Time Trend	Google Internet query share (IQS) data for gastroenteritis- related search terms correlated strongly with contemporaneous national and regional norovirus surveillance data in the United States.	6
Infectious Disease	Surveillance	Desai (2012) [70]	Use of Internet Search Data to Monitor Impact of Rotavirus Vaccination in the United States		To assess whether search engine data are able to capture the decline in rotavirus disease following vaccine implementation in the United States, using the United Kingdom, where routine rotavirus vaccination has not been implemented, as a control.	Not reported	United States and United Kingdom	January 1, 2004 to December 30, 2010	Google Insights	Not reported	rotavirus, rota virus, rotovirus, roto virus, rodo virus, rhoda virus	Y	Unclear	Y	Time Trend	Rotavirus IQS searches in the United States and United Kingdom correlated strongly with rotavirus laboratory detections, and prevaccine years in US model was less accurate than postvaccine years, when 1 week lag introduced to latter. Pre- and post- not as different for UK, and decline in rotavirus IQS and laboratory data in the United States after vaccine introduction are attributable to the effects of the vaccination program	8
Infectious Disease	Surveillance	Cho (2013) [71]	Correlation between National Influenza Surveillance Data and Google Trends in South Korea		To investigate the correlation between national influenza surveillance data and Google Trends in South Korea.	October 1, 2012	South Korea	September 9, 2007 to September 8, 2012	Google Trends	Not reported	new influenza, influenza, new flu, flu, swine flu, bird flu, bad cold, Tamiflu, fever, cough, sore throat (all in Korean), H1N1	N	N	Y	Time Trend	Google Trends for certain queries using the survey on influenza correlated with national surveillance data in South Korea, but it is insufficient for the use of predictive models.	0
Infectious Disease	Surveillance	Dukic (2011) [72]	Internet Queries and Methicillin-Resistant Staphylococcus aureus Surveillance		To assess the potential for Internet-based surveillance of methicillin-resistant Staphylococcus aureus and examine the extent to which it reflects trends in hospitalizations and news coverage.”	Not reported	United States	Not reported (2004 to 2008 in figure)	Google Trends	Not reported	MRSA, staph	N	N/A	Y	Time Trend	Google queries were a useful predictor of MRSA hospitalizations and explained 33% of quarterly variation when used alone. The correlation between the created model predictions and observed hospitalization rates was very high.	10
Infectious Disease	Surveillance	Valdivia (2010) [73]	Diseases Tracked by Using Google Trends, Spain		To explore whether this tool could be applicable for surveillance in non-English and non-French speaking countries and, more specifically, for Spain, expanding on the queries constructed by Pelat (2009) to include symptoms	August 2, 2009	Spain	January 2004 to February 2009	Google Insights	Not reported	gripe, gripe, aviar, vacuna, tos, neumonia, varicela	Y	N/A	Y	Time Trend	There is a good correlation between terms used and ILI and chickenpox.	24
Infectious Disease	Surveillance	Zhou (2011) [74]	Tuberculosis Surveillance by Analyzing Google Trends		To develop a syndromic approach to estimate the actual number of TB cases using Google search volume for the early detection of TB outbreaks.	May 20, 2009	United States	January 2004 to April 2009	Google Insights	Not reported	19 terms	N	N	Y	Time Trend	Disease related search volume has distinct temporal association with disease activity, resulting in a timely infectious disease surveillance system that can be updated every day, which is 12 weeks ahead of CD’s reports.	8
Infectious Disease	Surveillance	Zhou (2013) [75]	Monitoring epidemic alert levels by analyzing Internet search volume		To build a surveillance system using Google Trends for monitoring disease for epidemic alerts.	May 20, 2012	United States	January 2006 to December 2010	Google Trends	Y - health	47 terms	Y	N	Y	Time trend	The system proposed is able to predict disease alerts and provide real time surveillance results weeks before the CDC’s reports.	1
Mental Health and Substance Use	Causal Inference	Forsyth (2012) [76]	Virtually a drug scare: Mephedrone and the impact of the Internet on drug news transmission		To investigate whether news reports attributing harm to mephedrone precipitated increases in web-searches for the drug, and the nature and extent of online news stories concerning alleged mephedrone related deaths.	Not reported	Not reported (United Kingdom from introduction)	November 26, 2009 to November 26, 2010	Used both Google Trends and Google advanced search	Not reported	mephedrone	N/A	N/A	N	Time trend	The advent of the internet accelerated and inflated the mephedrone scare, but also that online media allowed web user generated information transmission, rather than simple dissemination by news media to audience.	16
Mental Health and Substance Use	Causal Inference	Sueki (2011) [22]	Does the volume of Internet searches using suicide-related search terms influence the suicide death rate: Data from 2004 to 2009 in Japan		To clarify the causal relationship between search volume and suicide death rate by examining the cross-correlation coefficient between the volumes of searches involving suicide-related search terms and the suicide death rate.	Not reported	Japan	January 2004 to December 2009	Google Insights	Y - All	Suicide, Depression, Suicide method	N	Unclear	Y	Time Trend	The volume of searches using the search terms suicide and suicide method are not correlated with the suicide death rate. A rising suicide death rate might be related to the increase in suicide-related search activity (particularly depression), but an increase in suicide-related search activity itself is not directly linked to the rise of suicide death rate.	10
Mental Health and Substance Use	Causal Inference	Ayers (2012) [77]	Novel surveillance of psychological distress during the great recession		To use Google Trends as a surrogate to assess population psychological distress in response to the recession.	Not reported	United States	2004 to 2010	Google Insights	Not reported	21 terms	Y	Unclear	Y	Time Trend	A 1% increase in mortgage delinquencies and foreclosures was associated with a 16% increase in psychological distress queries. Underemployment and unemployment showed effect too, but there is no effect of S&P and housing prices.	10
Mental Health and Substance Use	Causal Inference	Tefft (2011) [78]	To provide Insights on unemployment, unemployment insurance, and mental health using data from web searches		To simultaneously examine the effects of unemployment and unemployment Insurance on measures related to psychological distress.	Not reported	United States	2004 to 2009	Google Insights	Y - health	depression, anxiety	N	N/A	Y	Both	There was a positive relationship between the unemployment rate and the depression search index and a negative relationship between initial UI claims on the one hand and the depression and anxiety search indexes on the other. A lag analysis showed that an extended period of higher levels of continued UI claims is associated with a higher depression search index.	22
Mental Health and Substance Use	Causal Inference	Frijters (2013) [79]	Exploring the relationship between macroeconomic conditions and problem drinking as captured by Google searches in the US		To determine the relationship between unemployment and the relative frequency of Internet search for alcoholism at the level of US states during the last 5 years.	May 29, 2011	United States	January 2004 to April 2011	Google Insights	Y - health	alcohol, alcoholic, alcoholics, alcoholism, aa (and hotmail, cancer, yahoo)	Y	N/A	Y	Time Trend	The current recessionary period coincided with an almost 20% increase in alcoholism-related searches. Controlling for state and time effects, a 5% rise in unemployment is followed in the next 12 months by an approximately 15% increase in searchers.	3
Mental Health and Substance Use	Causal Inference	Bright (2013) [80]	Kronic hysteria: Exploring the intersection between Australian synthetic cannabis legislation, the media, and drug-related harm		To explore the relationship between media reports, policy responses, and drug-related harm from synthetic cannabis (Kronic), using Google Trends to identify volume of media stories published online and to outline the volume of searchers for these	Not reported	Australia	2011	Google Trends	Not reported	Synthetic Cannabis and Kronic	N	N	N	Time trend	Between April and June 2011, mentions of Kronic in the media increased. The number of media stories published online connected strongly with Google searches for the term Kronic.	9
Mental Health and Substance Use	Description	Yang (2010) [21]	Do Seasons Have an Influence on the Incidence of Depression? The Use of an Internet Search Engine Query Data as a Proxy of Human Affect		To investigate large-scale seasonal patterns of depression using Internet search query data as a signature and proxy of human affect.	Not reported	54 geographic areas worldwide (cities/metropolitan areas/states/provinces)	January 1, 2004 to June 30, 2009	Google Insights	Y - health	depression	N/A	N/A	Y	Time Trend	There was a seasonal trend of depression that was opposite between the northern and southern hemispheres, and this trend was significantly correlated with seasonal oscillations of temperature. The degree of correlation between searching for depression and temperature was latitude-dependent	28
Mental Health and Substance Use	Description	Hagihara (2012) [81]	Internet suicide searches and the incidence of suicide in young people in Japan		To examine the association between Internet suicide-related searches and the incidence of suicide in 20- and 30-year-old individuals in Japan.	October 10, 2009	Japan	January 2004 to May 2010	Google Insights	Not reported	A Suicide, Sites on suicide, Suicide methods, Hydrogen sulfide, Hydrogen sulfide suicide, Suicide hydrogen sulfide, Bulletin board system on Suicide, Suicide rates, Suicide by jumping, Depression suicide	Unclear	Unclear	Y	Time Trend	Internet searches using the terms hydrogen sulfide, hydrogen sulfide suicide, and suicide hydrogen sulfide at (t-11) were significantly related to the incidence of suicide among individuals 20–39 years old, and Internet searches using the terms BBS on suicide at (t-5) and suicide by jumping at (t-6) were significantly associated with the incidence of suicide among people in their 30 s.	15
Mental Health and Substance Use	Description	Gallagher (2012) [82]	5,6-Methylenedioxy-2-aminoindane: from laboratory curiosity to legal high’		To overview the current state of knowledge of MDAI and a critically analyze online information relating to its psychoactive effects, adverse reactions and use in combination with other drugs, as well as population interest in MDAI.	Not reported	Not reported (Worldwide, with focus on United Kingdom, United States, Germany, from results)	Not reported (2004–2010 from results)	Google Insights	Not reported	MDAI	N/A	N/A	N	Both	Internet-sourced products have been shown variously to contain mephedrone, and mixed compositions of inorganic substances, while containing no MDAI. Numbers of Internet searches have been considerably higher in the UK compared with Germany and the US.	11
Mental Health and Substance Use	Description	Steppan (2013) [83]	Are cannabis prevalence estimates comparable across countries and regions? A cross-cultural validation using search engine query data		To determine whether Cannabis-related search engine query data can be used as an external criterion to verify self-reported cannabis use	Not reported	Worldwide, restricted to countries with sufficient volume and ESPAD	2004 to 2011	Google Trends	Not reported	cannabis, ganja, grass, hashish, marijuana, purple haze, THC [Tetrahy- drocannabinol], weed, joint. The terms legalize and spice were also used as common interests in this field. For Italy, further local expressions were used: canna, cartine, erba, il fumo and spinello	N	N	Y	Cross Sectional	Google search index showed weaker associations with cannabis use than perceived availability.	1
Mental Health and Substance Use	Description	Song (2014) [84]	Psychological and social factors affecting Internet searches on suicide in Korea: a big data analysis of Google search trends		To identify factors related to searches on suicide in Korea; in particular, whether suicide rate by year and unemployment rate vary with the number of searches by month for stress, drinking, and exercise, reports of a celebrity suicide and searches for suicide	Not reported	Korea (compared with United States, United Kingdom and Australia)	January 1, 2004 to December 31, 2010	Google Trends	Not reported	suicide, stress, drinking and exercise. (Both English and Korean)	N	Unclear	N	Time Trend	Suicide and stress related searches are positively associated with suicide rates	0
Mental Health and Substance Use	Surveillance	Yang (2011) [85]	Association of Internet search trends with suicide death in Taipei City, Taiwan, 2004–2009		To evaluate the association between suicide and Internet trend data, and to identify search trend data that coincides or precedes the fluctuations in suicide death counts.	Not reported	Taipei City, Taiwan	Not reported (January 2004 to December 2009 in Figure)	Google Insights	Not reported	37 terms	Unclear	Unclear	Y	Time Trend	A set of suicide-related search terms, the trends of which either temporally coincided or preceded trends of suicide data, were associated with suicide death. Appropriate filtering and detection of potentially harmful sources in keyword-driven search results by search engine providers may be a reasonable strategy to reduce suicide deaths.	17
Mental Health and Substance Use	Surveillance	Gunn (2013) [86]	Using google searches on the internet to monitor suicidal behavior		To examine whether the association between suicide rates and Google searches for suicide are found over regions.	Not reported	United States (50 states)	2009	Google Trends	Not reported	commit suicide, suicide prevention, how to suicide	N	Unclear	N	Cross Sectional	Suicide rates for the 50 US states were positively associated with the search volume for all 3 terms (i.e. in states with higher suicide rates, there are higher volumes of searches of terms associated with suicide (according to 3 terms used).	4
Mental Health and Substance Use	Surveillance	McCarthy (2010) [87]	Internet monitoring of suicide risk in the population		To investigate the feasibility of monitoring the volume of suicide related internet searches as a tool to more rapidly identify trends that could influence suicide risk on a population-wide level	May 1, 2009	United States	2004 to 2009. Correlation with CDC 2004–2007.	Google Trends	Not reported	Suicide, teen suicide, depression, divorce, unemployment	N	Unclear	Y	Time Trend	Google search volumes correlated to CDC statistics for both suicide and self-injury, but in patterns that differed by age. Whereas internet search activity was negatively correlated to the suicide rate in the general population, it was positively correlated to both intentional self-injury and completed suicide among youth.	32
Mental Health and Substance Use	Surveillance	Bragazzi (2013) [88]	A Google Trends-based approach for monitoring NSSI		To investigate NSSI queries trends and patterns.	Not reported	Italy	2004 to 2012	Google Trends	Not reported	autolesionismo correlated with suicide, blood, cutting, razor, depression, anxiety, bullying, anorexia, bulimia	N	N/A	N	Both	The pattern of Internet search volume revealed a cyclic trend and a regular pattern. Statistically significant scores for autocorrelation and significant correlations.	0
Mental Health and Substance Use	Surveillance	Page (2011) [89]	Surveillance of Australian suicidal behaviour using the Internet?		To determine whether internet searches using Google in Australia relating to ‘ways to commit suicide’ showed any seasonal trends or were related to unemployment rates, and could be used to for surveillance of Australian suicidal behaviour.	Not reported	Australia	February 2004 to March 2011	Google Insights	Not reported	how to commit suicide, ways to kill yourself, suicide pact, suicide hanging	Y	Unclear	Y	Time Trend	Trends in Internet searches of suicide terms using Google are not a sufficiently straightforward indicator of the levels of suicidal behavior in Australia and showed no seasonality, with limited evidence for an association with unemployment trends.	6
Mental Health and Substance Use	Surveillance	Yin (2012) [90]	Monitoring a toxicological outbreak using Internet search query data		To determine whether internet search query data could have been used as a surveillance method for the “bath salts” outbreak.	October 2, 2011	United States	July 2010 to February 2011	Google Insights	Not reported	bath salts (soap and methamphetamine as controls)	N/A	Unclear	Y	Both	GIS data for the search term “bath salts” was correlated with exposures to bath salts reported to US poison centers over the study period, and poison center exposures and GIS data did not differ significantly in detecting a change from the baseline. There was also an association when comparing exposures by state to search volumes by state for “bath salts”.	7
																	

### Classification of Published Google Trends Articles

#### Topic Domain

We classified articles by the primary topic addressed by each article. By consensus we identified four main topic domains: infectious diseases (27% of articles), mental health and substance use (24%), other non-communicable diseases (16%), and general population behavior (33%). The general population behavior category included all health-related behaviors excluding mental health and substance use.

#### Study aim

There were three categories of study aim: causal inference (27%), description (39%), and surveillance (34%). We defined *causal inference* studies as those in which the primary aim was to evaluate a hypothesized causal relationship with Google Trends data. An example of a causal inference study is Ayers et al. (2014), who used search queries to explore the potential link between a public figure’s cancer diagnosis and population interest in primary cancer prevention. We defined *descriptive* studies as those that aimed to describe temporal or geographic trends and general relationships, without reference to a hypothesized causal relationship. An example of a descriptive study is Stein et al. (2013), who assessed public interest in LASIK surgery and how levels of interest have changed over time in the United States and other countries. A particular subset of descriptive studies were *surveillance* studies, which we defined as those in which the stated aim was to evaluate the use of Google Trends to predict or monitor real-world phenomena. An example of a surveillance study is Desai et al. (2012), who assessed whether Google search trends are appropriate for monitoring Norovirus disease.

### Methodology of Published Google Trends Articles

#### Documentation of Search Strategy

Table 3 summarizes the documentation of search strategy. Only 34% of articles documented the date the search was conducted.

**Table 3:** Summary of Methodology Documentation.

	Percent of Articles Documenting Information (N)
Date of Search	**34%** (24/70)
Google Data Source	**99%** (69/70)
**Query Variables**	
Location of Search	**87%** (61/70)
Time Period of Search	**87%** (61/70)
Query Category	**19%** (13/70)
**Search Input Syntax**	
Clear Search Input, total’	**39%** (27/70)
Excluding studies with only one term (n = 8)	**31%** (19/62)
Specifics:	
Use of combination of terms (55/70 Eligible’)	
Yes	**31%** (17/55)
Unclear	**18%** (10/55)
No	**51%** (28/55)
Use of Quotes (52/70 Eligible∼)	
Yes	**0%** (0/52)
Unclear	**81%** (42/52)
No	**19%** (10/52)
**Overall Reproducibility**	
Reproducible Articles*	**7%** (5/70)
**Rationale**	
Rationale Provided for Search Input	**67%** (47/70)

’defined as providing clear use of quotes and combination.

‘eligibility = use of more than one term, ex. HIV + AIDS.

∼eligibility = use of a term with more than one word, ex. “HIV epidemic”.

*defined as addressing those fields which are modifiable within the portal by the user (therefore excluding date of search and data source).

Of the variables that can be manipulated within the portal, within the methods section of the papers, 87% documented the location searched, 87% documented the time period searched, and only 19% clearly stated the query category used.

With respect to the search input, only 39% provided a clear search input. Excluding studies with only a single, one-word search term, which are not eligible for using quotations or combinations of terms, only 31% provided a clear search input. Of the articles eligible for using quotations (search terms with >1 word), 81% were unclear and 19% did not use quotations; none provided a clear use of quotations. Of the articles eligible for using a combination of terms (>1 search term used), 31% used a combination, 18% were unclear, and 51% used individual terms.

#### Reproducibility and Rationale

Overall, only 7% of articles provided requisite documentation for a reproducible search strategy within their methods section; among original articles alone it was 8%.

In addition, we found that only 67% of articles provided a rationale for their search input.

#### Analytic Method

Time trend analysis (comparisons across time periods) was used by 70% of the studies, cross-sectional analysis (comparisons across different locations at a single time period) by 11% of studies, and both by 19%. A variety of analytic methods were used in conjunction with Google Trends output data, including correlation, continuous density hidden Markov models, ANOVA, Box–Jenkins transfer function models, t-tests, autocorrelation, multivariable linear regression, time series analyses, wavelet power spectrum analysis, Cosinor analysis, and the Mann-Whitney test.

### Subanalysis of Surveillance Articles and Validation

Among the 24 surveillance studies, 71% used time trend analysis, 25% cross-sectional analysis, and 4% both. Among articles using time trend analysis, 33% utilized lead-time analysis (using Google Trends data from a specific time point/interval to predict events occurring at a later time). Overall, 17% of studies used training/testing data sets and 13% had a time horizon (time period over which surveillance was assessed) <1 year. More detailed information can be found in Table S1.

With respect to validation, 92% made comparisons against external datasets to validate the Google Trends output; the remaining 8% did not validate their findings. Examples of sources of comparison datasets include disease prevalence data from centers such as the United States Centers for Disease Control, drug revenues from shareholder reports of pharmaceutical companies, and unemployment rates from the Australian Bureau of Statistics.

There was a wide range of correlation statistics, from 0.04 to 0.98 (Figure S3). Among the 15 papers that used Pearson product-moment correlation, 80% had at least one correlation statistic greater than 0.70.

### Checklist for the Documentation of Google Trends Use

In view of the limitations of existing studies identified during this review, we developed a checklist to improve the quality and reproducibility of studies that use Google Trends in the future (Table 4). This was created based directly on the variables that can be manipulated within the Google Trends portal, differences in which could provide differing results among researchers, and the need to provide search strategy rationale. A hypothetical example of a “well-documented” search strategy is included within Table 4. Of note, we used brackets to separate the search input from the body text to ensure that the reader understands what was searched for with clear syntax; similar approaches of segregation might be used.

**Table 4:** Checklist for Documentation of Google Trends.

Section/Topic	#	Checklist Item	Reported on Page #
Search Variables			
Access Date	1	Provide the date(s) when Google Trends was accessed and when the data was downloaded.	
Time Period	2	Identify all the time periods that were searched for in Google Trends, providing up to the Month and Day in detail.	
Query Category	3	Identify which query category was used for search; if not using a query category, designate that “all query categories were used”, which is the default setting.	
**Search Input**			
Full Search Input	4	Provide the full search input(s) that were queried for in Google Trends, along with the appropriate documentation of search syntax (detailed in 4a and 4b). Ensure that the provision of the search input is clear, using brackets (as in the example below) or other delineators to separate the search input from the body text.	
Combination	4a	If more than one search term was used, document whether those terms were used in combination with a plus sign (+), or if terms were excluded with a minus sign (-). If terms were not used in combination, state so clearly.	
Quotation Marks	4b	If there was more than one word in any search term (ex. “lipid guideline”), document whether those words were queried with quotation marks or not.	
**Rationale for Search Strategy**			
For Search Input	5	Provide the reasoning behind the choice of search input.	
For Settings Chosen	6	Provide the reasoning for the settings/search variables chosen to specify the search.	
**Hypothetical Example**			
On May 1, 2014, we queried Google Trends and downloaded the data for the following search input: [“cholesterol guideline” + “lipid guideline” + “cholesterol recommendation” + “statin recommendation”]. We searched within the United States from January 1, 2013 to May 1, 2014 using the “health” query category. We chose these search terms based on a survey of cardiovascular disease patients’ most likely search terms for this topic. We chose January 1, 2013 as the start date to capture baseline interest in the year before the publication (November 2013), chose United States because it is the country of the guideline publication, and chose the “health” query category because we wanted to assess interest in the context of health.			

## Discussion

In this systematic review of the use of Google Trends in healthcare research, we found that researchers are increasingly utilizing the tool in a diversity of areas in myriad ways; these articles are being widely cited. Furthermore, the majority of surveillance studies validated Google Trends output against external datasets and many had strong correlation statistics. However, the majority of studies lack thorough documentation of search methodologies, which precludes the reproducibility of results; less than 10% of articles are reproducible. In addition, search rationale is often not provided. Thus, while the data within Google Trends holds promise, significant variability and limitations remain around study quality and reliability.

The 70 papers included in our review reflected a wide variety of topics and uses. A large proportion of articles used Google Trends to investigate population behavior, which is a logical application of the tool given its basis in user searches. The large proportion of infectious disease articles may stem from the precedent set by Google Flu Trends.[3] Nearly equal numbers of studies used Google Trends for causal inference, surveillance, and description, demonstrating the ability to use the tool to answer a range of questions. There was an increase in publications over time, and the median citation rate (7 per article) is comparable to the average for all scientific articles (7.64 per article).[12] These observations suggest increasing awareness of and the leveraging of information from the tool. Locations studied using the tool were geographically widespread, particularly outside of the United States where conventional data collection may be challenging and resource intensive. Nevertheless, there is evidence of a positive results publication bias, which may be due to the novelty of the tool and authors not submitting and/or editors not accepting negative – and, therefore, perhaps uninteresting – results.[13], [14] This publication bias also may be due to researchers retroactively constructing hypotheses about interesting findings after the results are known for a given Google Trends experiment, which can be fast and easy to conduct.[15].

Despite the potential insights and research opportunities that Google Trends provides, many problems were observed with the documentation of methodology. Thorough documentation is necessary to ensure the reproducibility and replicability of the results, which are fundamental tenets of good science.[16] The inability to reproduce studies in the sciences has been well-documented, and it serves as a central problem to the utility and credibility of research.[17], [18] Yet, in our study, only 7% of articles provided clear documentation of the necessary fields to be reproducible. This is especially salient for using Google Trends given the many search fields and multiple options available within each field. Researchers may not have known how to document their methods since this is still a nascent tool for research, without guidance or methodological standards for its use from either Google Inc. or the research community. Furthermore, there were particular issues with the clarity of search inputs. For example, it was often unclear whether quotations provided for a search term were actually used in the search input or were merely given to distinguish the term from the rest of the text. A potential reason for varying presentation of search input syntax may be that possible search syntaxes may have changed over time.[8], [19] Nevertheless, the poor documentation of methods also raises larger questions about researchers using Google Trends without knowing the ways in which the tool can be operated.

Different selections of terms to address a common question with Google Trends can produce disparate results and conclusions, and providing the rationale behind these selections is necessary for a reader to better understand the study methods and to increase the face validity of the study.[20] Yet, studies often provided no rationale for their search input. For instance, we do not know why studies chose a given selection of terms or used a specific syntax. Furthermore, there are larger questions about the search strategy as a whole, such as why certain query categories and dates for searching were chosen. Nevertheless, certain studies demonstrated more thorough search strategies and strong rationales for search inputs, particularly accounting for the basis of Google Trends data in user searches. For instance, Desai et al. (2012) included potential misspellings of their search words to fully capture a specific search pattern. In addition, Cho et al. (2013) developed their search inputs by surveying their population of interest, in which they inquired about what search terms subjects would have used to search for influenza. Similar strategies could be adopted by future studies to ensure that their search terms accurately capture the outcome of interest. More guidance is needed by Google to assist researchers in how to produce an optimal search strategy to answer a given question.

Over 90% of surveillance studies compared Google Trends with established data sets, which were often trusted sources of surveillance data. A large number of correlation studies had moderate to strong strengths of association, which demonstrates the potential of Google Trends data to be used for the surveillance of health-related phenomena. For example, Jena et al. (2013) found a strong correlation between searches for HIV and US CDC HIV incidence rates, and were able to construct a model based on searches from years 2007–2008 to accurately predict state HIV incidence for 2009–2010. Moreover, Samaras et al. (2012) showed that Google Trends could have been used to forecast the peak of scarlet fever in the UK 5 weeks before its arrival. Although studies are promising, strong correlations alone do not support the use of Google Trends for surveillance, and further work is needed to substantiate the reliability and real world applicability of Google Trends as a tool to monitor health-related phenomena.

In light of our results, we have proposed a basic checklist for the documentation of Google Trends use. This checklist can serve as a baseline standard to ensure methodological understanding and reproducibility by researchers who choose to use the tool in the future.

While this checklist is a good step forward to improve the reproducibility of results by researchers, there are still larger limitations in the Google Trends tool itself. We cannot clearly ascertain user characteristics and intent from search data, which limits the ability to draw generalizable conclusions about population search behavior. In addition, Google Trends captures the search behavior of only a certain segment of the population – those with Internet access and using Google Search instead of other search engines. However, the major limitation of Google Trends is the lack of detailed information on the method by which Google generates this search data and the algorithms it employs to analyze it. Furthermore, temporal changes in the interface and capabilities of the Google Trends over time are not documented, which may lead to variation in the search output and therefore study findings.

Moving forward, several steps can be taken to improve the verification of Google Trends study results and the reliability of the tool for research, both on the part of the independent researcher and Google Inc. Researchers should strive to make the raw data they downloaded from the Google Trends available online (as Yang et al., 2010 did [21]) and create an archive or screenshot of the website as they searched it (as Sueki et al., 2011 did [22]) to provide transparency of their methodology and encourage open science with this open tool. Researchers could also evaluate the methods and results of others and themselves to check if there is consistency over time. We encourage Google Inc. to provide a chronology of important changes to Google Trends – in the past and to come – to put researchers’ methods and findings in context. Furthermore, if Google Trends continues to be used for research purposes, a discussion and collaboration between Google Inc. and the research community is necessary to create a set of best practices to ensure that the tool is being used responsibly and that its tremendous potential to derive meaningful insights from population search behavior could be fully harvested. While full transparency may not be possible due to commercial sensitivities, informed guidance is needed to ensure the conduct of ethical science. For example, Google Inc. could work together with groups of researchers to detail how to construct optimal queries to fully take advantage of the algorithms at work and to improve the tool to increase the quality of research. In addition, it is important to remember that these conclusions apply not only to Google Trends, but also other similar tools, which currently exist or will likely emerge from existing data sources, that are not intrinsically designed to be utilized for research. In a Big Data era where information and technologies, particularly those that are readily accessible to the public and research community, are growing, mindfulness must be paid to their application in scientific research and efforts must be made to ensure the conduct of good science. One must look no further than the recent controversy around the reliability of Google Flu Trends data to predict influenza incidence and the lack of transparency and inability to verify its results.[23].

Our study has certain limitations. First, given the diversity of topics and uses, there are inherent challenges in the classification of articles. However, at least two independent abstractors reviewed each article and category of abstraction, and disagreements were resolved by group consensus. Second, there are no prior standards to evaluate literature from novel data sources such as Google Trends. Third, our assessment of Google Trends was based on the current syntactic possibilities, but they may have changed over time.[8], [19] Conversely, this supports our concerns about undocumented changes to the tool. Finally, there is a possibility that we had an incomplete retrieval of Google Trends articles in our search strategy. However, we conducted an extensive, systematic search of two databases, in addition to reviewing article references, to capture as many articles as possible. Notably, our study focused on the evaluation of the use of Google Trends in research, and we refrained from making any commentary about the conclusions drawn by researchers in these studies. Further studies are needed to rigorously evaluate the interpretations of causal inference studies and the validity of Google Trends for surveillance.

## Conclusion

Google Trends holds potential as a free, easily accessible means to access large population search data to derive meaningful insights about population behavior and its link to health and health care. However, to be reliably utilized as a research tool, it would have to be more transparent, which will increase the trustworthiness of both the results generated and its general applicability for health care research. Furthermore, researchers must make efforts to clearly state their rationale and document their experiments to ensure the reproducibility of results. The lessons gleaned from this review are also instructive for other tools not intrinsically designed for research that may emerge in an era of Big Data to ensure that they are used appropriately by the scientific community.

## Supporting Information

Appendix S1 **MEDLINE Search Strategy.**
(DOCX)

Table S1 **Surveillance Variable Abstraction Results.**
(DOCX)

Figure S1 **Google Trends Web Page Output.** Screenshot of a Google Trends search output when queried for 3 terms: [“Google Trends”], [“Google Insights”], and [“Google Trends” + “Google Insights”]. We searched Worldwide, using all query categories, for the time period from January 2004 to March 2014 (site accessed: 3/17/14).
(TIFF)

Figure S2 **Distribution of Articles Included in Our Review by Year of Publication.** Notably, we did not include those articles published in 2014 (n = 5) in the figure, as they represent only part of that year.
(TIFF)

Figure S3 **Forrest Plot of Measures of Association for Surveillance Studies Using Pearson’s Correlation.** Plot of correlation statistics from each surveillance study that used Pearson’s correlation. For studies with multiple correlation statistics, each was plotted individually.
(TIFF)

Checklist S1 **PRISMA Checklist.**
(TIFF)
