# Supplementary material for: The Use of Google Trends in Health Care Research: A Systematic Review
Source: PLoS One. 2014 Oct 22;9(10):e109583. doi: 10.1371/journal.pone.0109583 (PMC4215636; doi:10.1371/journal.pone.0109583)
Supplement: Appendix S1 — MEDLINE Search Strategy. (DOCX) [file pone.0109583.s001.docx]

**APPENDIX S1. MEDLINE Search Strategy** (Date: January 3, 2014)

| **#** | **Searches** | **Results** |
| --- | --- | --- |
| 1 | exp Search Engine/ | 712 |
| 2 | exp Internet/ | 54084 |
| 3 | google.tw. | 3031 |
| 4 | google trend$.tw. | 14 |
| 5 | google insight$.tw. | 25 |
| 6 | (google adj3 trend$).tw. | 46 |
| 7 | (google adj3 insight$).tw. | 25 |
| 8 | (google adj3 search$).tw. | 952 |
| 9 | (google adj3 portal$).tw. | 5 |
| 10 | internet search$.tw. | 946 |
| 11 | online search$.tw. | 457 |
| 12 | ((web or online or internet) adj3 brows$).tw. | 997 |
| 13 | (search$ adj3 volum$).tw. | 128 |
| 14 | exp Behavioral Risk Factor Surveillance System/ | 1289 |
| 15 | exp Population Surveillance/ | 53261 |
| 16 | exp Epidemiological Monitoring/ | 3953 |
| 17 | exp Sentinel Surveillance/ | 5002 |
| 18 | 1 or 2 or 14 or 15 or 16 or 17 | 111848 |
| 19 | 3 or 4 or 5 or 6 or 7 or 8 or 9 or 10 or 11 or 12 or 13 | 5328 |
| 20 | 18 and 19 | 1823 |
| 21 | limit 20 to humans | 1320 |
| **22** | **limit 21 to english language** | **1249** |
