# Supplementary material for: The Use of Google Trends in Health Care Research: A Systematic Review
Source: PLoS One. 2014 Oct 22;9(10):e109583. doi: 10.1371/journal.pone.0109583 (PMC4215636; doi:10.1371/journal.pone.0109583)
Supplement: Table S1 — Surveillance Variable Abstraction Results. (DOCX) [file pone.0109583.s002.docx]

**Table S1. Surveillance Variable Abstraction Results**

| **Author (Year)** | **Title** | **Measure/**  **Outcome^1^** | **Analysis Type** | **Time division** | **Geographic division** | **Source** | **Search term(s)** | **Measure of association^3^** | **Magnitude of relationship (and p-value or confidence interval, if reported)** | **Real-time or lead-time (amount of lead time in parentheses)** | **Used separate training and testing datasets for validation (if yes, details in parentheses** | **Time horizon** |
| --- | --- | --- | --- | --- | --- | --- | --- | --- | --- | --- | --- | --- |
| Zheluk (2013) | Internet Search Patterns of Human Immunodeficiency Virus and the Digital Divide in the Russian Federation: Infoveillance Study | HIV (prevalence) | Cross Sectional | N/a | Sub-national (Russia) | Russian Federal AIDS Center | Russian translations of a) HIV b) AIDS | Spearman correlation coefficient | a) 0.672 (p=0.004) b) 0.584 (p≤0.001) | Real-time | No | 2011 |
| Dukic (2011) | Internet Queries and Methicillin-Resistant Staphylococcus aureus Surveillance | MRSA | Time Trend | Quarterly | National (US) | Hospital discharge data from the University HealthSystems Consortium Clinical Database | Multiple (MV) | Correlation | 0.93 (p<0.001) | Real-time | No | 2004-2008 |
| Kang (2013) | Using Google Trends for Influenza Surveillance in South China | 1. Influenza-life illness (ILI) (clinical diagnosis) | Time Trend | Weekly | Sub-national (Guangdong province) | 56 sentinel clinics | Chinese translations of a) Fever b) H1N1 | Correlation | a) 0.73 (95% CI: 0.66, 0.79) b) 0.51 (95% CI: 0.26, 0.69) | Real-time | No | 2008-2011 |
|  |  | 2. Laboratory-confirmed influenza |  |  |  | 22 laboratories | Chinese translation of Influenza A |  | 0.64 (95% CI: 0.43, 0.79) |  |  |  |
| Samaras (2012) | Syndromic surveillance models using Web data: The case of scarlet fever in the UK | Scarlet fever | Time Trend | Weekly | National (UK) | UK National Health Agency weekly reports | Multiple (MV) | Correlation | 0.86 (year 2008); 0.97 (year 2009); 0.89 (year 2010) | Lead-time (five weeks) | No | 2008-2010 |
| Cho (2013) | Correlation between National Influenza Surveillance Data and Google Trends in South Korea | 1. Influenza-life illness (ILI) (clinical diagnosis) | Time Trend | Weekly | National (South Korea) | 850 sentinel clinics | Korean translation of H1N1 | Correlation | 0.53 (p<0.05) | Real-time | No | September 2007 - September 2012 |
|  |  | 2. Laboratory-confirmed influenza |  |  |  | 91 laboratories | Korean translation of bad cold |  | 0.33 (p<0.05) |  |  |  |
| Althouse (2011) | Prediction of Dengue Incidence Using Search Query Surveillance | Dengue fever (clinical and laboratory-confirmed) | Time trend | Weekly | National (Singapore) | Singapore Ministry of Health | Multiple (MV) | Correlation | 0.931 | Both (assessed 0 and 4 week lead times) | Yes (trained on 2004-2010 data, tested on 2011 data) | 2005–2011 |
|  |  |  |  | Monthly | City (Bangkok, Thailand) | Thai Bureau of Epidemiology |  |  | 0.869 |  |  |  |
| Desai (2012) | Norovirus Disease Surveillance Using Google Internet Query Share Data | 1. Gastroenteritis (ED visits) | Time Trend | Monthly | Regional (greater Boston) | Prospective citywide syndromic surveillance data from the Boston Public Health Commission | Multiple (CSV) | R-squared | 0.74 | Lead-time (two weeks) | No | 1) January 2006 - July 2011 |
|  |  | 2. Gastroenteritis (hospitalizations) |  |  | National | Nationwide Inpatient Sample |  |  | 0.95 (between January 2007 and May 2010); 0.70 (Between January 2004 and May 2007) | Real-time |  | 2) January 2004 - June 2007 |
| Walcott (2011) | Determination of geographic variance in stroke prevalence using Internet search engine analytics | Stroke (prevalence) | Cross Sectional | N/a | State (US) | CDC MMWR | Multiple (CSV) | Correlation | 0.47 (p = 0.0005) | Real-time | No | 2005-2010 |
| Zhou (2013) | Monitoring epidemic alert levels by analyzing Internet search volume | 1. Hepatitis A | Time trend | Weekly | National (US) | CDC | Multiple (MV) | "Error rate" (how often model failed to predict the categorized incidence, or "alert level") | 8.1% | Real-time | Yes (tested model in every week t using model that was continuously updated using data from all weeks t < 1) | 2006-2010 |
|  |  | 2. Hepatitis B |  |  |  |  |  |  | 1.8% |  |  |  |
| Gunn (2013) | Using Google searches on the internet to monitor suicidal behavior | Suicide | Cross Sectional | N/a | State (US) | McIntosh, 2012^2^ | a) “Commit suicide” b) "how to suicide" c) "suicide prevention" | Correlation | a) 0.31 b) 0.21 c) 0.61 | Real-time | No | 2009 |
| Zhou (2011) | Tuberculosis Surveillance by Analyzing Google Trends | Tuberculosis | Time Trend | Weekly and quarterly | National (US) | CDC | Multiple (MV) | Mean squared error | 4.18×10^5^ (a priori estimation); 5.31×10^5^ (a posteriori estimation) | Real-time | Yes (tested model in every week t using model that was continuously updated using data from all weeks t < 1) | January 2004 - April 2009 |
| McCarthy (2010) | Internet monitoring of suicide risk in the population | 1. Suicides | Time Trend | Annual | National (US) | CDC Web-based Injury Statistics Query and Reporting System | "Suicide" | Correlation | -0.900 (general population); 0.699 (youth) | Real-time | No | 2004-2009 |
|  |  | 2. Intentional self-injury |  |  |  |  |  |  | -0.0436 (general population); 0.498 (youth) |  |  |  |
| Schuster (2010) | Using Search Engine Query Data to Track Pharmaceutical Utilization: A Study of Statins | Lipitor global revenues | Time Trend | Annual | Worldwide | Pfizer Shareholder Reports | "Lipitor" | Correlation | 0.98 (p<0.001) | Real-time | No | 2004-2009 |
| Willard (2013) | Internet Search Trends Analysis Tools Can Provide Real-time Data on Kidney Stone Disease in the United States | Kidney stones (prevalence) | Cross Sectional | N/a | State (US) | Second Cancer Prevalence Study | "Kidney stones" | R-squared | 0.25 (p<0.001) | Real-time | No | January 2004 - April 10 2010. |
| Yang (2011) | Association of Internet search trends with suicide death in Taipei City, Taiwan, 2004-2009 | Suicide deaths | Time Trend | Monthly | City (Taipei City, Taiwan) | Department of Health, Taiwan | Chinese translations of a) major depression b) divorce (MV) | Partial correlation | a) 0.512 (p<0.001) b) 0.319 (p=0.008) | a) Real-time b) lead-time (two months) | No | January 2004 - December 2009 |
| Breyer (2011) | Use of Google Insights for Search to Track Seasonal and Geographic Kidney Stone Incidence in the United States | Nephrolithiasis (hospitalizations) | Time Trend | Monthly | National (US) | Nationwide Inpatient Sample (NIS) | "Kidney stones" | Correlation | 0.81 (p=0.0014) | Real-time | No | January 2006 - December 2007 |
| Valdivia (2010) | Diseases Tracked by Using Google Trends, Spain | 1. Influenza-like illness (ILI) | Time Trend | Weekly | National (Spain) | Spanish National Epidemiology Center | Spanish translations of: a) influenza b) influenza (-avian - vaccine c) cough d) pneumonia | Correlation | a) 0.70 b) 0.81 c) 0.74 d) 0.84 | Lead-time (two weeks) | No | January 2004 - February 2009 |
|  |  | 2. Chickenpox |  |  |  |  | Spanish translation of chickenpox |  | 0.96 |  |  |  |
| Pelat (2009) | More Diseases Tracked by Using Google Trends | 1. Influenza-like illness (ILI) | Time Trend | Weekly | National (France) | French Sentinel network | French translations of: a) influenza (-avian -vaccine) b) influenza | Correlation | a) 0.82 (p<0.001) b) 0.34 (p<0.001) | Real-time | No | January 2004 - February 2009 |
|  |  | 2. Acute diarrhea |  |  |  |  | French translation of gastroenteritis |  | 0.9 |  |  |  |
|  |  | 3. Chickenpox |  |  |  |  | French translation of chickenpox |  | 0.78 | Lead-time (one week) |  |  |
| Page (2011) | Surveillance of Australian suicidal behaviour using the Internet? | Unemployment rates ("a known correlate of suicidal behaviour in the Australian population") | Time Trend | Monthly | National (Australia) | Australian Bureau of Statistics | Multiple (CSV) | Beta coefficient (from Unobserved Component Model) | 0.26 (p =0.077) | Real-time | No | February 2004 - March 2011 |
| Bragazzi (2013) | A Google Trends-based approach for monitoring NSSI | None ("Complete epidemiological data for NSSI are not available or are largely incomplete") | Cross Sectional | N/a | National (Italy) | N/a | N/a | N/a | N/a | N/a | No | 2004-2012 |
| Desai (2012) | Use of Internet Search Data to Monitor Impact of Rotavirus Vaccination in the United States | 1. Rotavirus | Time Trend | Weekly | National (US) | 25 US sentinel laboratories | Multiple (CSV) | Correlation | 0.88 (p<0.001) | Real-time | No | 2004-2010 |
|  |  | 2. Rotavirus |  |  | National (UK) | 187 UK sentinel laboratories |  |  | 0.80 (p<0.001) |  |  |  |
| Yin (2012) | Monitoring a toxicological outbreak using Internet search query data | 1. Exposures to bath salts | Time-trend | Weekly | National (US) | National Poison Database System | "Bath salts" | Correlation | 0.84 | Real-time | No | July 2010 - February 2011 |
|  |  | 2. Exposures to bath salts | Cross-sectional | N/a | State (US) |  |  |  | 0.79 |  |  |  |
| Jena (2013) | Predicting New Diagnoses of HIV Infection Using Internet Search Engine Data | HIV | Cross Sectional | N/a | State (US) | CDC | "HIV" | Correlation | 0.83 (p<. 001) | Real-time | Yes (trained on 2007–2008 data, tested on 2009–2010 data) | 2007-2010 |
| Carneiro (2009) | Google Trends: A Web-Based Tool for Real-Time Surveillance of Disease Outbreaks | None | Time-trend | N/a | N/a | N/a | N/a | N/a | N/a | N/a | No | January 2004 - March 2009 |
| **Footnotes:** | |  |  |  |  |  |  |  |  |  |  |  |
| ^1^ Incidence measures, unless otherwise specified | | |  |  |  |  |  |  |  |  |  |  |
| ^2^ McIntosh, J.L. (for the American Association of Suicidology) 2012. U.S.A. suicide: 2009 official final data. Washington, DC: American Association of Suicidology, dated January 12, 2012, downloaded from 〈http://www.suicidology.org〉 | | | | |  |  |  |  |  |  |  |  |
| ^3^ "Correlation" refers to Pearson correlation, unless otherwise specified | | |  |  |  |  |  |  |  |  |  |  |
| MV: individual term search volumes combined in multivariate model | | |  |  |  |  |  |  |  |  |  |  |
| CSV: using combined search volume | | |  |  |  |  |  |  |  |  |  |  |
