# Supplementary figures and images for: The Use of Google Trends in Health Care Research: A Systematic Review
Source: PLoS One. 2014 Oct 22;9(10):e109583. doi: 10.1371/journal.pone.0109583 (PMC4215636; doi:10.1371/journal.pone.0109583)

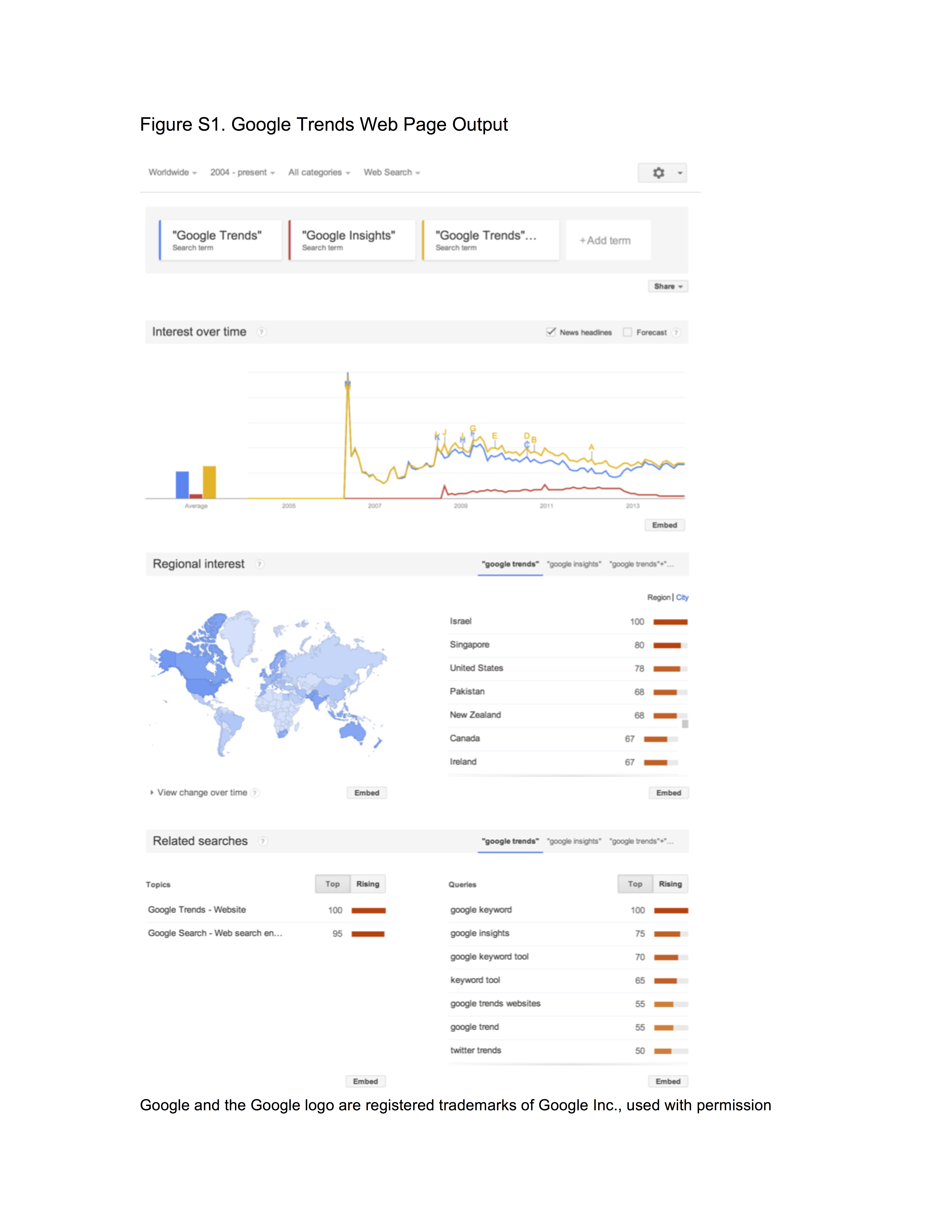

Supplement: Figure S1 — Google Trends Web Page Output. Screenshot of a Google Trends search output when queried for 3 terms: [“Google Trends”], [“Google Insights”], and [“Google Trends” + “Google Insights”]. We searched Worldwide, using all query categories, for the time period from January 2004 to March 2014 (site accessed: 3/17/14). (TIFF) [file pone.0109583.s003.tiff]

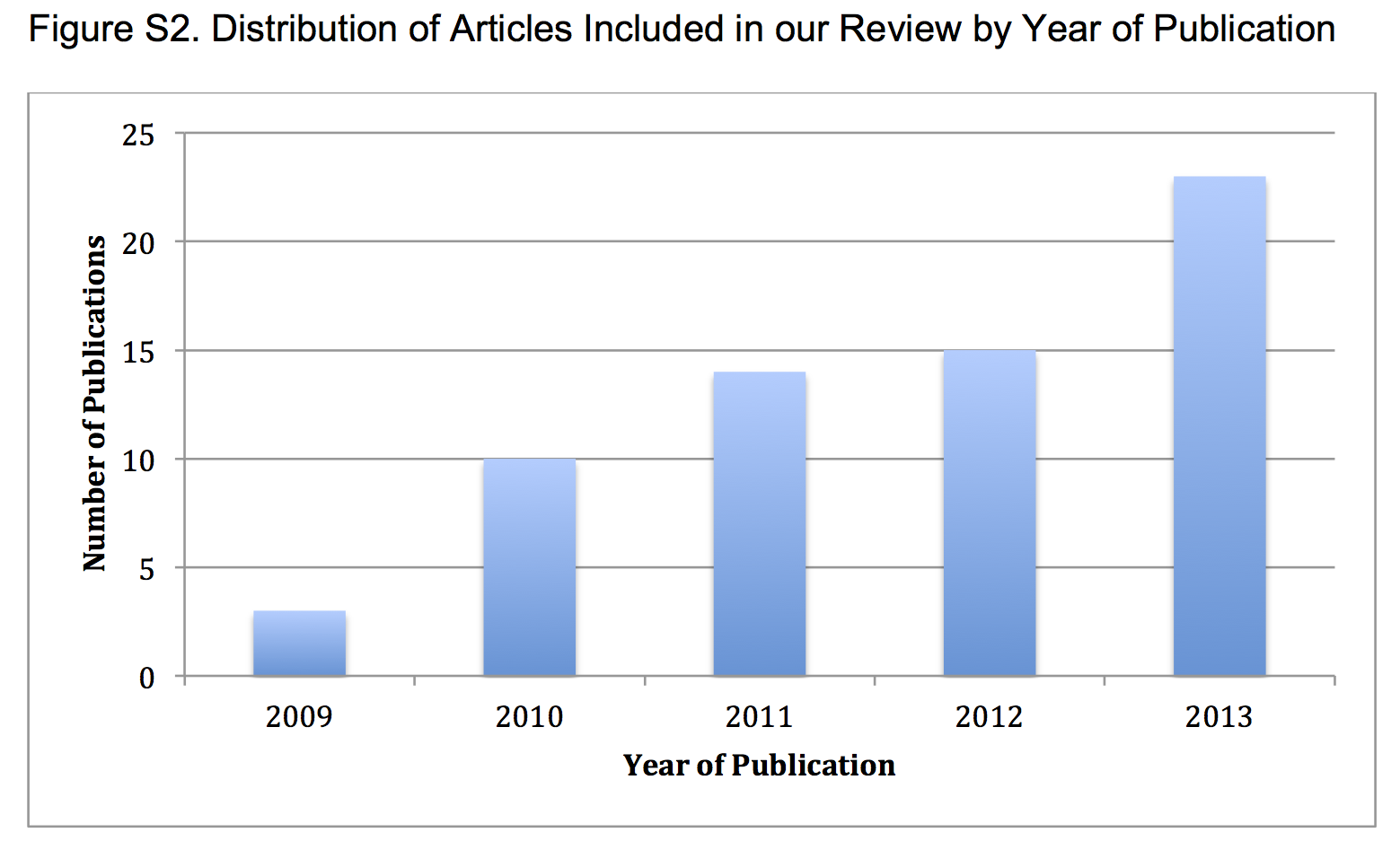

Supplement: Figure S2 — Distribution of Articles Included in Our Review by Year of Publication. Notably, we did not include those articles published in 2014 (n = 5) in the figure, as they represent only part of that year. (TIFF) [file pone.0109583.s004.tiff]

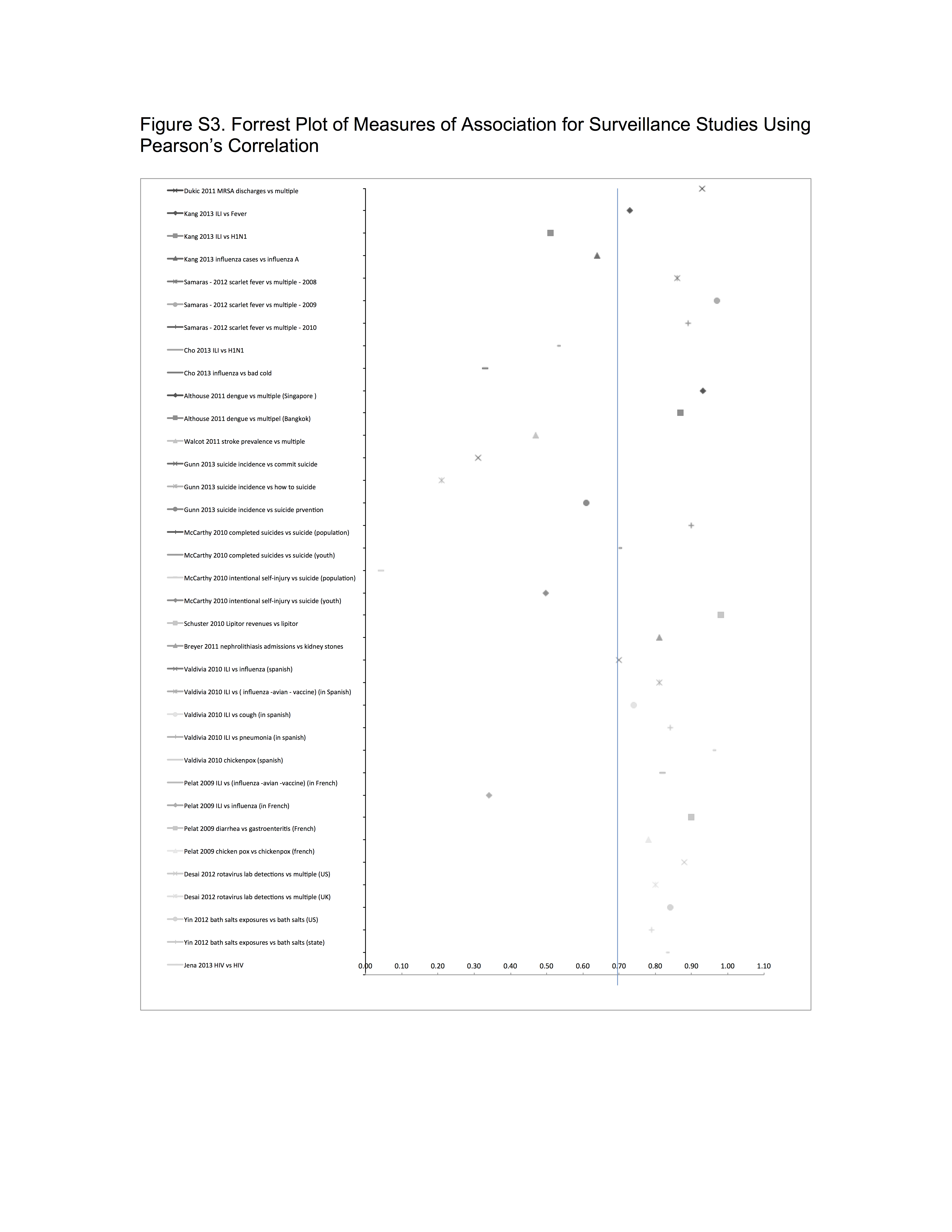

Supplement: Figure S3 — Forrest Plot of Measures of Association for Surveillance Studies Using Pearson’s Correlation. Plot of correlation statistics from each surveillance study that used Pearson’s correlation. For studies with multiple correlation statistics, each was plotted individually. (TIFF) [file pone.0109583.s005.tiff]

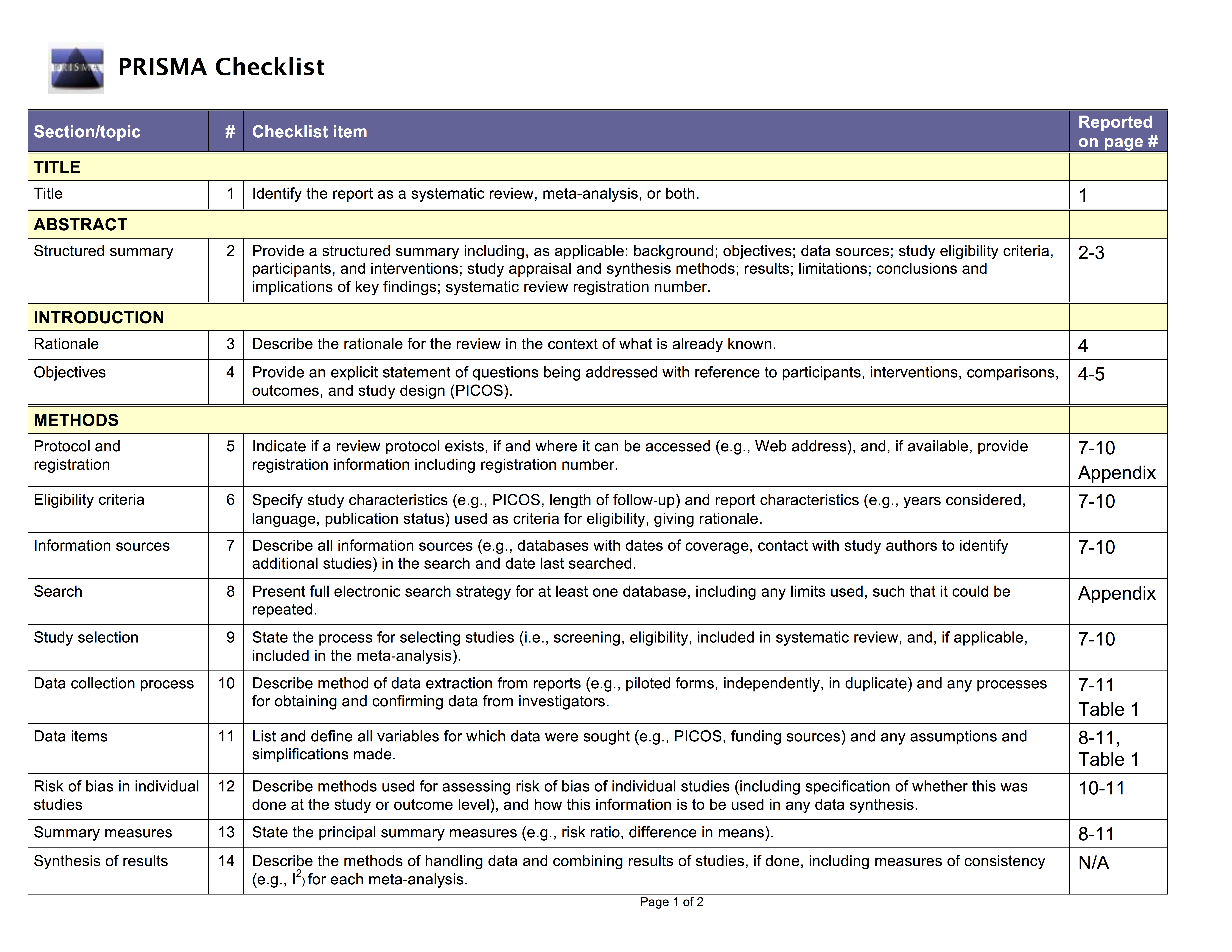

Supplement: Checklist S1 — PRISMA Checklist. (TIFF) [file pone.0109583.s006.tiff]
